# Beta cell–targeted PD-1 agonist inhibits cell-mediated autoimmunity in pancreas tissue slices

**DOI:** 10.1126/sciadv.aec9029

**Published:** 2026-04-01

**Authors:** Matthew W. Becker, Matthew E. Brown, Katherine Wiseman, Ana Laura Chiodetti, Mollie K. Huber, Alexandra E. Cuaycal, Pumin Sintara, Sandra M. Ferreira, Dylan Smurlick, Jessie M. Barra, Andrew M. Ladd, Denise M. Drotar, Mark A. Atkinson, Peter Weber, Hussein Al-Mossawi, Holger A. Russ, Tara M. Mahon, Todd M. Brusko, Giovanna Bossi, Edward A. Phelps

**Affiliations:** ^1^ J. Crayton Pruitt Family Department of Biomedical Engineering, Herbert Wertheim College of Engineering, University of Florida, Gainesville, FL, USA.; ^2^ Department of Pathology, Immunology, and Laboratory Medicine, College of Medicine, University of Florida, Gainesville, FL, USA.; ^3^ University of Florida Diabetes Institute, University of Florida, Gainesville, FL, USA.; ^4^ Immunocore Ltd., Abingdon, UK.; ^5^ Department of Infectious Diseases and Immunology, College of Veterinary Medicine, University of Florida, Gainesville, FL, USA.; ^6^ Department of Pharmacology & Therapeutics, College of Medicine, University of Florida, Gainesville, FL, USA.; ^7^ Department of Pediatrics, College of Medicine, University of Florida, Gainesville, FL, USA.; ^8^ Department of Biochemistry and Molecular Biology, College of Medicine, University of Florida, Gainesville, FL, USA.

## Abstract

This research evaluates a therapeutic approach based on tissue-targeted immunomodulation with a potential broad application to treat autoimmune diseases including type 1 diabetes (T1D). We generated a bispecific immune agonist that binds beta cells and suppresses autoreactive T cells. These bispecific molecules called immune modulating monoclonal–T cell receptor (TCR) against autoimmune disease (ImmTAAI), consist of a human-specific TCR–targeting domain fused with a programmed death-1 agonist. We used live pancreas slices to demonstrate targeting of ImmTAAI molecules to preproinsulin peptide–HLA-A2 complexes on human beta cells. ImmTAAI molecules protected beta cells from T cell killing by increasing T cell motility and inhibiting effector molecule and cytokine secretion. ImmTAAI treatment also increased the motility of islet-infiltrating T cells in slices from a donor with recent-onset T1D and preserved insulin secretion in slices cocultured with T cell avatars transduced with diabetogenic TCRs. These data demonstrate that ImmTAAI molecules have the potential to limit T cell activity locally, making this an attractive platform to elicit targeted immunoregulation in T1D.

## Teaser

A beta cell–targeting, affinity-enhanced TCR fused to a PD-1 agonist inhibits cellular immunity relevant to human type 1 diabetes.

## INTRODUCTION

Type 1 diabetes (T1D) is characterized by the selective destruction of insulin-producing beta cells by autoreactive T cells (*1*–*3*). Approaches to manipulate mechanisms of central and peripheral tolerance are actively being explored to treat T1D autoimmunity (*4*–*8*); however, systemic immune suppression carries negative drawbacks for patient health. The polyclonal autoimmune response in T1D, which at the time of diagnosis involves T and B cells recognizing multiple epitopes from an incompletely defined list of islet antigens, poses a challenge for the clinical success of antigen-specific immunotherapies (*9*–*12*). One possible solution would involve the design of therapies that promote islet-localized tolerance by targeting beta cells. Such a strategy would interface with the entire repertoire of islet-invading T cells without requisite knowledge of the autoantigen subset while avoiding the need for systemic immune suppression.

Inhibitory receptor agonists represent a class of drugs targeting peripheral immune tolerance mechanisms, which are beginning to make their way into clinical trials for autoimmune diseases (*13*). Of the inhibitory receptors, programmed death-1 (PD-1) and its ligand, PD-L1, are particularly relevant to T1D (*14*). PD-1 is expressed on infiltrating lymphocytes in nonobese diabetic (NOD) mice (*15*) and in human T1D organ donors (*16*). Disrupting PD-1/PD-L1 interactions with a monoclonal antibody leads to accelerated T1D in mice (*17*–*19*). Furthermore, administration of clinically approved cancer therapies that disrupt PD-1/PD-L1 interactions can lead to the development of autoimmune diabetes (*20*–*22*). Positive responses to drug therapies for T1D in clinical trials are also correlated with T cell exhaustion markers such as PD-1 (*23*, *24*). Since PD-1 signaling in T cells depends on T cell receptor (TCR) engagement with peptide–human leukocyte antigen (pHLA) complexes (*25*–*28*), therapeutics that target tissue-specific pHLA may enable selective activation of PD-1 signaling in disease-relevant tissues, thereby promoting localized immune tolerance.

We previously designed a bispecific PD-1 agonist immune modulating monoclonal TCR against autoimmunity (ImmTAAI) molecule with a targeting end composed of a soluble TCR that is highly specific for a preproinsulin (PPI) peptide, PPI_15–24_, which is bound to HLA-A*02:01 (HLA-A2) on beta cells, together with an effector end composed of an agonistic PD-1 binding nanobody (*29*). PD-1 agonist ImmTAAI molecules are potent inhibitors of effector T cells and reduce cytokine secretion and beta cell killing in vitro (*29*). T cell inhibition only occurs when PD-1 agonist ImmTAAI molecules are bound to target cells, an activity that does not occur when ImmTAAI molecules are in solution (*29*). However, these studies were performed with a homogeneous beta cell line; hence, further investigation is needed to understand the scope of bispecific ImmTAAI molecules in complex primary human tissues. Pancreas tissue slices are one tool that can help conduct such studies. These explants are thin slices (~120-μm thickness) of live tissue from transplant-quality donor organs embedded in agarose and maintained in a functional state in vitro (*30*, *31*). Each tissue slice contains both exocrine and endocrine tissue with intact islets capable of glucose sensing and insulin secretion (*32*, *33*). Slices also preserve islets in the native tissue context in disease states such as T1D where the islet may be infiltrated and structurally compromised (*33*–*35*).

In this study, we investigated whether ImmTAAI molecules maintain beta cell and HLA-A2 specificity within the heterogeneous tissue of pancreas slices. Through immunofluorescent labeling and confocal microscopy of live tissue, we observed that ImmTAAI molecules maintain specificity with minimal to no off-target binding. ImmTAAI molecules also protected beta cells from killing by T cell avatars transduced with diabetogenic TCRs. ImmTAAI demonstrated effective binding to beta cells in a recent-onset T1D donor, resulting in changes to T cell motility and reducing T cell:beta cell interactions, suggesting that pancreas slices can be used to assess thisl bispecific beta cell–targeted therapy, which has the potential to treat T1D.

## RESULTS

### Fluorescently labeled ImmTAAI molecules bind target pHLA and inhibit TCR signaling

**Fig. 1.**
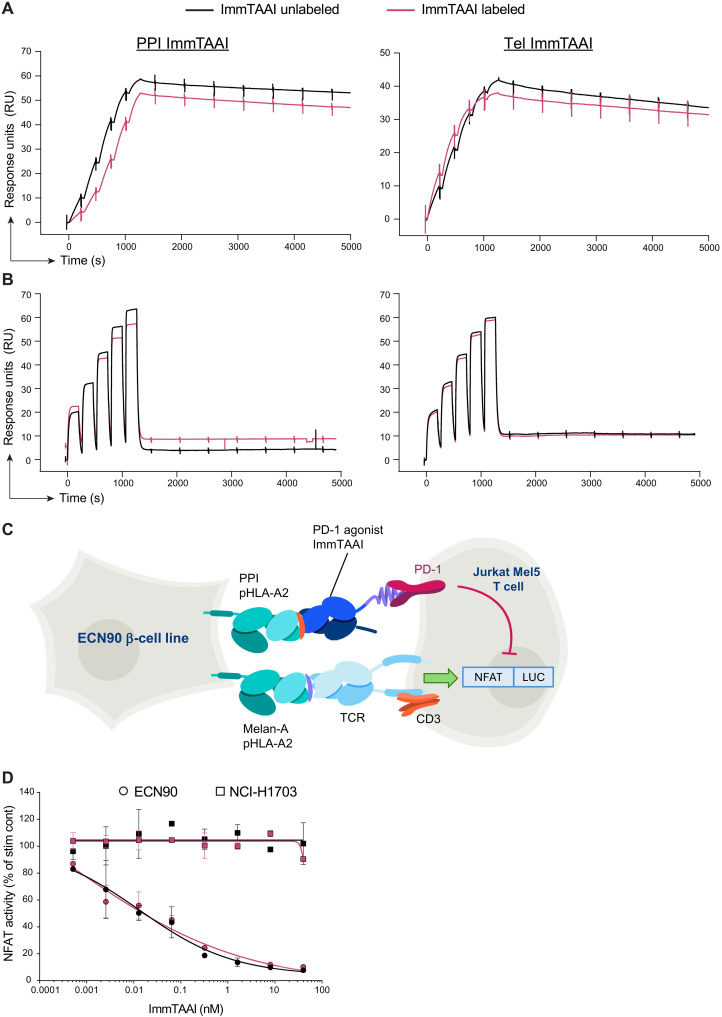
Fluorescently labeled ImmTAAI molecules bind target pHLA and inhibit TCR signaling. (**A**) CF647-labeled ImmTAAI molecules were assessed for pHLA binding and compared to unlabeled molecules by SPR using BIAcore 8K. Binding to PPI or Tel cognate pHLA was carried out at 37°C. (**B**) Similarly, CF647-labeled ImmTAAI molecules were assessed for PD-1 binding and compared to unlabeled molecules. PD-1 binding was carried out at 25°C. (**C**) Schematic of the ECN90 beta cell line: Jurkat NFL Mel5 PD-1 reporter assay. (**D**) ECN90 or NCI-H1703 cells were pulsed with Melan-A–activating peptide, and titrations of unlabeled or labeled PPI ImmTAAI molecules were added.

We generated cyanine-based far-red fluorescent dye (CF647)–labeled ImmTAAI molecules specific for PPI peptide PPI_15–24_ pHLA-A2 (PPI ImmTAAI) and for human telomerase reverse transcriptase (hTERT) peptide hTERT_540–548_ pHLA-A2 (Tel ImmTAAI, negative control) to visualize binding in live pancreas slices. We conducted surface plasmon resonance (SPR) studies to determine binding kinetics for labeled and unlabeled PPI ImmTAAI and Tel ImmTAAI to their corresponding surface-immobilized cognate pHLA. We obtained single-cycle kinetics profiles, where a series of five ImmTAAI concentrations were sequentially injected over immobilized pHLA, followed by a long dissociation phase (Fig. 1A). Both labeled and unlabeled PPI ImmTAAI demonstrated similar affinity for cognate pHLA in the picomolar range, with the time taken for half the bound ImmTAAI to dissociate from pHLA (*t*_1/2_) > 6 hours (Fig. 1A). Similarly, both labeled and unlabeled Tel ImmTAAI retained picomolar affinity for cognate pHLA with *t*_1/2_ > 4 hours (Fig. 1A). Hence, ImmTAAI molecules strongly bind their cognate pHLA, and pHLA binding was not substantially affected by the CF647 fluorescent tag.

Next, we assessed the effect of CF647 labeling on ImmTAAI effector region binding to PD-1. We obtained single-cycle kinetics profiles where a series of five PD-1 concentrations were sequentially injected over immobilized ImmTAAI. The anti–PD-1 portion of the ImmTAAI molecule displayed nanomolar affinity with a relatively short *t*_1/2_ of ~13 s (Fig. 1B). Binding to PD-1 was likewise unaffected by labeling with CF647 for both PPI and Tel ImmTAAI molecules (Fig. 1B).

To identify any potential impact of CF647 labeling on cellular potency or selectivity, the labeled and unlabeled PPI ImmTAAI molecules were also tested in a Jurkat NFL Mel5 PD-1 reporter assay (Fig. 1C) (*29*). This incorporated the ECN90 human beta cell line as targets with natural PPI pHLA-A2 presentation and NCI-H1703 human squamous cells that do not present the PPI epitope. Target cells were pulsed with the melanoma antigen (Melan-A) to engage the Mel5 TCR on the Jurkat cells and evaluate the inhibitory effect of PD-1 engagement by the targeted ImmTAAI on T cell activity. Both labeled and unlabeled PPI ImmTAAI molecules inhibited cellular TCR signaling with comparable potency (Fig. 1D). No inhibitory activity at ImmTAAI concentrations up to 40 nM was observed when using NCI-H1703 target cells (Fig. 1D). Overall, these data demonstrate that the CF647 labeling did not affect ImmTAAI potency or selectivity.

### ImmTAAI binding and labeling of beta cells in pancreas slices is concentration dependent

**Fig. 2.**
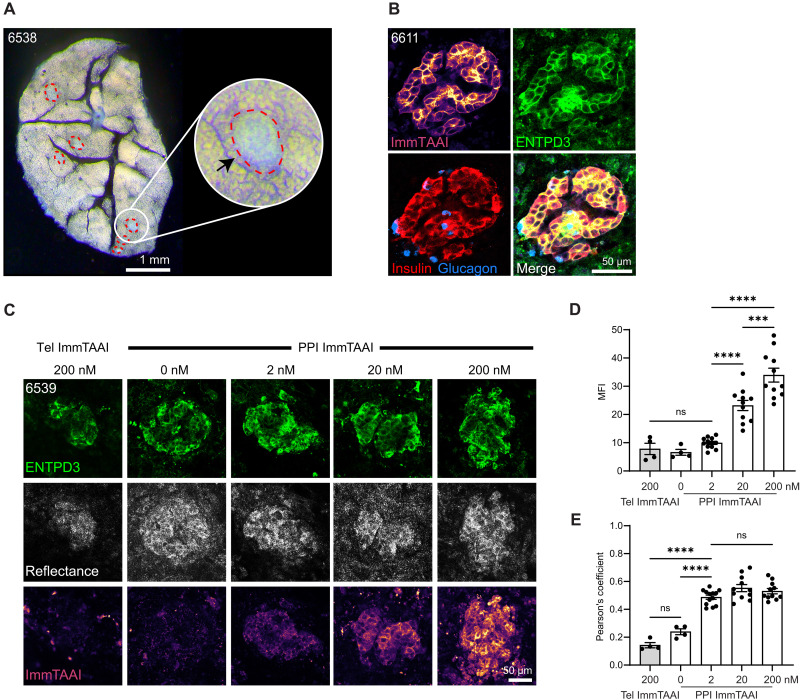
ImmTAAI binding and labeling of beta cells in pancreas slices is concentration dependent. (**A**) Dark-field stereomicroscopy image of a pancreas slice showing islets outlined in red. (**B**) Pancreas slice incubated with CF647-labeled PPI ImmTAAI and subsequently fixed and stained for insulin, glucagon, and ENTPD3, and imaged by confocal. (**C**) Confocal microscopy images of live pancreas slices incubated with CF647-labeled PPI ImmTAAI at 2, 20, or 200 nM for 1 hour and stained with a labeled primary antibody for cell surface ENTPD3. The off-target CF647-labeled Tel ImmTAAI was used as a control. (**D**) MFI quantification of confocal microscopy images showing the dose-dependent binding of PPI ImmTAAI to tissue slices. (**E**) Pearson’s coefficient colocalization analysis of ImmTAAI fluorescent signal with anti-ENTPD3 fluorescent signal. PPI ImmTAAI colocalizes with anti-ENTPD3 well at all three concentrations tested, indicating that beta cell–specific binding is maintained in native tissue. Individual dots represent measurements per islet (means from one donor for Tel ImmTAAI and two donors for PPI ImmTAAI labeled). Statistical differences were determined by two-way analysis of variance (ANOVA) followed by Tukey’s post hoc analysis. ns, not significant; ***P < 0.001, ****P < 0.0001.

Live tissue slices of 120-μm thickness were generated by the Network for Pancreatic Organ donors with Diabetes (nPOD) program (*36*, *37*) from human pancreas organ donors using a vibratome. Donor information is listed in table S1. Pancreas slices contained islets in the native tissue microenvironment as visualized by dark field stereomicroscopy (Fig. 2A). Pancreas slices from a control donor without diabetes expressing HLA-A2 were incubated with CF647-labeled PPI ImmTAAI, fixed, and stained for insulin, glucagon, and the beta cell surface marker ectonucleoside triphosphate diphosphohydrolase-3 (ENTPD3) (Fig. 2B) (*35*, *38*–*40*). ImmTAAI colocalized with ENTPD3 in the plasma membrane of beta cells. Next, we incubated live slices from an HLA-A2–positive donor without diabetes with CF647-labeled ImmTAAI molecules at different concentrations for 1 hour and imaged the slices by live-cell confocal microscopy (Fig. 2C and fig. S1). Islets in live slices were located using a combination of reflected light backscattered off the insulin granules (*41*) and costaining for ENTPD3. The control Tel ImmTAAI did not label the tissue. Incubation with 2, 20, and 200 nM PPI ImmTAAI resulted in islet-specific labeling with a dose-dependent increase in mean fluorescence intensity (MFI) (Fig. 2D). PPI ImmTAAI colocalized with ENTPD3 starting at 2 nM, whereas the weak fluorescent signal detected from Tel ImmTAAI or unlabeled slices did not colocalize with ENTPD3 (Fig. 2E). Image quantification for MFI showed that 20 nM ImmTAAI was required to achieve significant increases in islet-localized ImmTAAI brightness relative to control or unlabeled samples due to frequent autofluorescent background spots in the islet tissue, which we attribute to accumulated lipofuscin in the long-lived postmitotic beta cells (*42*). Incubation with ImmTAAI molecules did not negatively affect cell viability in slices (fig. S1). Together, these data indicate that PPI ImmTAAI binds specifically to beta cell surfaces in a concentration-dependent manner in the complex environment of live native pancreatic tissue.

### ImmTAAI binding is HLA specific in tissue slices

**Fig. 3.**
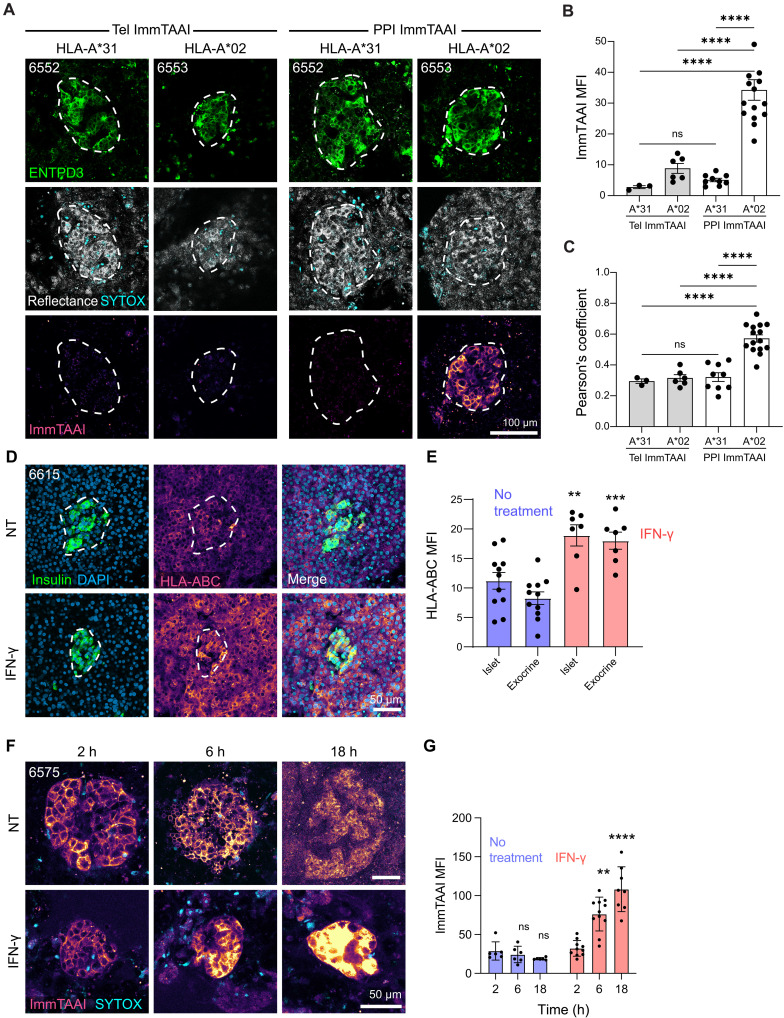
ImmTAAI binding is HLA specific in tissue slices. (**A**) Confocal microscopy images of pancreas slices incubated with 20 nM CF647-labeled PPI ImmTAAI for 2 hours (h) and costained for ENTPD3. No ImmTAAI staining is observed in slices from HLA-A*31 tissue. Islet granularity (reflectance) and viability (SYTOX blue) are also shown. (**B**) MFI analysis of ImmTAAI binding in HLA-A2–negative and –positive tissue, showing binding only in HLA-A2–positive tissue. (**C**) Pearson’s coefficient colocalization analysis of ImmTAAI fluorescent signal with anti-ENTPD3 fluorescent signal. ImmTAAI signal colocalizes with ENTPD3 only in HLA-A2–positive tissue. Each dot represents a separate islet from one or two separate slices per condition (*n* = 1 donor for Tel ImmTAAI in HLA-A*31 tissue, *n* = 2 donors for HLA-A2) and is the mean of three to six *z*-planes taken per islet. (**D**) Confocal microscopy images of pancreas slices treated with a 5-hour pulse of IFN-γ (100 IU/ml) and then fixed after 24 hours and stained for insulin and HLA-ABC. (**E**) MFI analysis of HLA-ABC expression in islet and exocrine regions. Each dot represents a separate islet/exocrine region image from ≥three slices per condition. (**F**) Live confocal microscopy images of pancreas slices treated with a 5-hour pulse of IFN-γ (100 IU/ml) and incubated with 20 nM CF647-labeled PPI ImmTAAI at 2, 6, or 18 hours. (**G**) MFI analysis of ImmTAAI signal at each time point post–IFN-γ treatment. Each dot represents a separate islet from ≥three slices per condition. NT, no treatment. Statistical differences were determined by two-way ANOVA followed by Tukey’s post hoc analysis. ns, not significant. **P < 0.01, ***P < 0.001, ****P < 0.0001.

The TCR domain of ImmTAAI molecules specifically targets PPI_15–24_ presented in the context of HLA-A2. To confirm the HLA specificity of the ImmTAAI in primary tissue, we compared its binding in pancreatic slices from an HLA-A2–positive donor and an HLA-A2–negative donor (HLA-A*31). Tissue slices were incubated with 20 nM CF647-labeled PPI or Tel ImmTAAI for 2 hours and labeled with anti-ENTPD3 before live imaging (Fig. 3A). There was no specific fluorescent signal in tissue from the HLA-A*31 donor for either PPI or Tel ImmTAAI, while significant binding occurred with PPI ImmTAAI in the HLA-A2 donor (Fig. 3B). We again confirmed that this binding was beta cell specific by quantifying colocalization with AF594-labeled anti-ENTPD3 (Fig. 3C).

T1D is characterized by HLA class I hyperexpression within islets (*43*, *44*). HLA class I expression can be induced in pancreatic slice cultures by treatment with exogenous interferon-γ (IFN-γ) (*45*). Thus, we pretreated slices with a pulse of IFN-γ (100 U/ml) for 5 hours, washed off the cytokine, and assessed islets after 18 hours for HLA class I up-regulation. Immunostaining of fixed slices confirmed a robust up-regulation of HLA class I in both islet and exocrine tissues (Fig. 3, D and E). ImmTAAI binding accumulated over time following IFN-γ treatment (Fig. 3, F and G). Together, these results show that ImmTAAI binding to beta cell surfaces depends on cell surface expression of pHLA, and IFN-γ–mediated inflammation results in increased ligation of ImmTAAI on the surface of beta cells.

### PPI ImmTAAI suppresses T cell engagement with beta cells to prevent cell-mediated killing

**Fig. 4.**
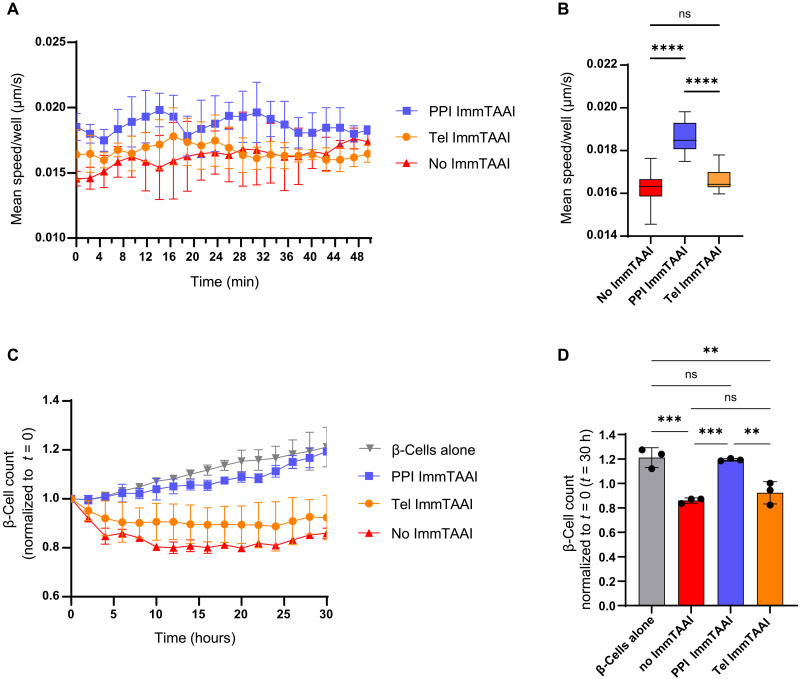
PPI ImmTAAI suppresses T cell engagement with beta cells to prevent cell-mediated killing. (**A**) Time series line plot representing the mean speed per well of T cell clone 4b (PPI_6–14_ specific) in the presence of EndoC-βH2 red cells, 10 nM PPI ImmTAAI, 10 nM Tel ImmTAAI, or without molecule. (**B**) Boxplot representing the mean speed per well of all the time points shown in (A) (ns, not significant; ****P < 0.0001; one-way ANOVA with multiple comparisons analyzed by Tukey’s test). Data are representative of three independent experiments. (**C**) Growth curves of EndoC-βH2 red cells in the presence of T cell clone 4b at 1:1 E to T ratio, 10 nM PPI ImmTAAI, 10 nM Tel ImmTAAI, or no molecule over time. Time series line plot of live EndoC-βH2 red cell number over time normalized to the initial cell count at time = 0. (**D**) Bar plots representing the mean per well ± SD of live EndoC-βH2 red cells count at time = 30 hours normalized to the initial cell count at time = 0. Statistical differences were determined by one way ANOVA with multiple comparisons analyzed by Tukey’s test. ns, not significant. **P < 0.01, ***P < 0.001. Data are representative of three independent experiments.

Antigen recognition by T cells results in swarming behavior and T cell arrest on target cells in a manner dependent on TCR engagement (*46*). The PD-1/PD-L1 pathway has been implicated in promoting immunological tolerance by suppressing the TCR-driven signal to stop migrating (*18*, *19*). PD-1/PD-L1 complexation reduces the interaction time and lowers cytokine production when CD4^+^ T cells engage with antigen-presenting cells (*47*). Conversely, blockade of PD-1/PD-L1 interactions decreases T cell migration velocity, enhances cytokine production, and boosts TCR signaling and activation (*19*). Therefore, PD-1 agonist ImmTAAI bound to beta cells could increase the mobility of T cells and protect beta cells from autoreactive T cell–mediated killing. To measure this effect, migration velocity was tracked in PPI_6–14_–specific T cells cocultured with EndoC-βH2 beta cells. The PPI_15–24_ ImmTAAI targeted to beta cells enhanced the mobility of T cells by reducing the time of interaction between T cells and target cells (Fig. 4, A and B), while the untargeted Tel ImmTAAI molecule did not affect T cell speed as compared to the no ImmTAAI molecule condition (Fig. 4, A and B). To determine whether there is a correlation between T cell mobility and beta cell killing, the beta cell viability was assessed over time (Fig. 4, C and D). As previously reported (*29*), PPI ImmTAAI suppressed T cell killing and preserved beta cell growth in vitro (Fig. 4, C and D). Beta cell protection was not detected with untargeted Tel ImmTAAI or in the absence of ImmTAAI (Fig. 4, C and D). To exclude the possibility that the observed reduced T cell killing is not due to steric or competitive interference at the beta cell surface, two control molecules were tested. One consisted of the PPI TCR alone, and the second was the PPI TCR fused to an irrelevant VHH antibody specific for the actin-like protein 8 (ACTL-8), an intracellular component of the cytoskeleton. While the PPI ImmTAAI prevents beta cell killing by T cells, neither the PPI ACTL-8 molecule nor the PPI TCR had any protective effect on beta cells (fig. S2). Together, the PPI ImmTAAI by delivering PD-1 agonism specifically to beta cells suppressed T cell function and enhanced T cell mobility preventing beta cell destruction.

### PPI ImmTAAI reduces T cell stopping in live pancreas slices from a recent-onset T1D donor

**Fig. 5.**
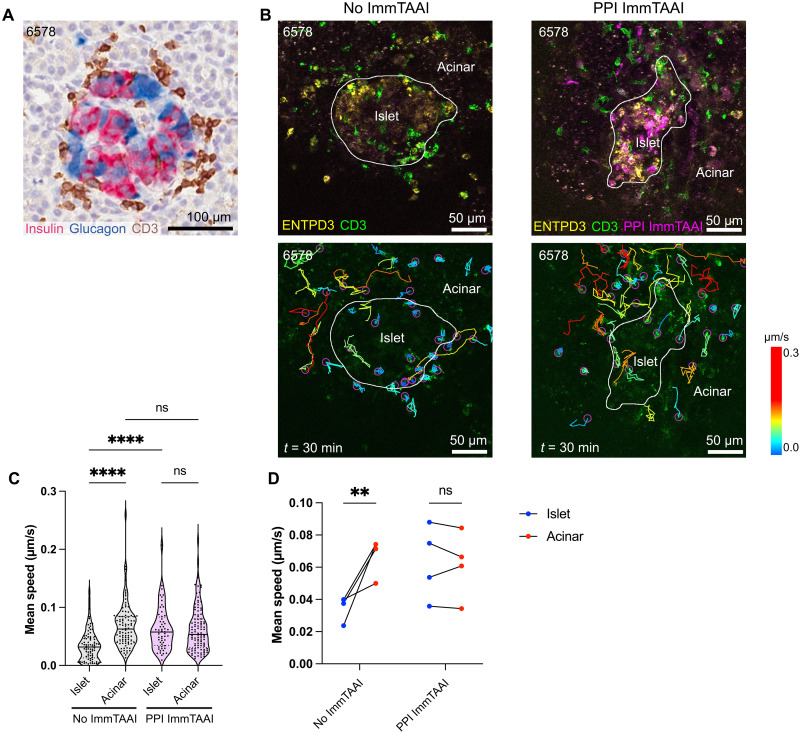
PPI ImmTAAI reduces T cell stopping in live pancreas slices from a recent-onset T1D donor. (**A**) Representative immunohistochemistry image from the nPOD histopathology database (https://portal.jdrfnpod.org/) of an insulitic islet from at-onset T1D donor 6578. (**B**) Still frames from live-cell confocal microscopy videos depicting ENTPD3^+^ islets with CD3^+^ infiltrates with and without ImmTAAI (top) and corresponding T cell tracks over 30 min. (**C**) Quantification mean speed of total T cell tracks inside and outside of the islet boundary [white outline in (B)] pooled from four islet recordings each with and without ImmTAAI treatment; two-way ANOVA with multiple comparisons analyzed by Tukey’s test. (**D**) Average T cell speed per islet recording inside and outside of the islet boundary with and without ImmTAAI treatment. Statistical differences were determined by two-way ANOVA with multiple comparisons analyzed by Tukey’s test. ns, not significant. **P < 0.01, ****P < 0.0001.

To confirm that the PPI ImmTAAI maintains its beta cell–binding capacity in donors with recently diagnosed T1D, pancreas slices from an HLA-A2–positive donor with recently diagnosed T1D (nPOD 6551, 20 years of age, 0.58 years T1D duration; table S1) and the remaining beta cells were labeled with 20 nM CF647-labeled PPI ImmTAAI for 2 hours and costained for ENTPD3. We observed robust ImmTAAI binding to islets in slices from this donor (fig. S3). Although nPOD donor 6551 was reported to have insulitis in some islets [nPOD histopathology database and (*35*)], we were unable to record live slices with insulitis and ImmTAAI labeling in this donor, consistent with the variable intensity and frequency of insulitis that is well-documented in human T1D (*3*, *48*). We identified a second HLA-A2–positive donor with new-onset T1D (nPOD 6578, 11 years of age, 0 years T1D duration; table S1), whose islets had both high levels of T cell infiltration and remaining beta cells (Fig. 5A). We treated slices with 20 nM CF647-labeled PPI ImmTAAI overnight, stained endogenous T cells with an antibody for CD3 and beta cells with an antibody for ENTPD3, and then measured the effect of the ImmTAAI on CD3^+^ T cell–infiltrated islets. We generated 30-min time lapse recordings by confocal microscopy to track T cell motility (Fig. 5B). In slices not treated with ImmTAAI, we observed many T cells with low motility within the islet boundary, while T cells outside of the islet had high motility (Fig. 5, C and D). The behavior of the slow-moving T cells, which remained within a confined localization, is suggestive of specific TCR-HLA class I interactions promoting T cell stopping, consistent with our previously reported observations in T1D slice donors (*35*). In slices treated with PPI ImmTAAI, the mean T cell speed within islets matched that of the acinar tissue (Fig. 5, C and D). These data suggest that PD-1 ligation by beta cell surface–bound ImmTAAI molecules increases the motility of the endogenous T cell infiltrate in islets of a donor with recent-onset T1D.

### PPI ImmTAAI suppresses killing of sBCs by T cell avatars

**Fig. 6.**
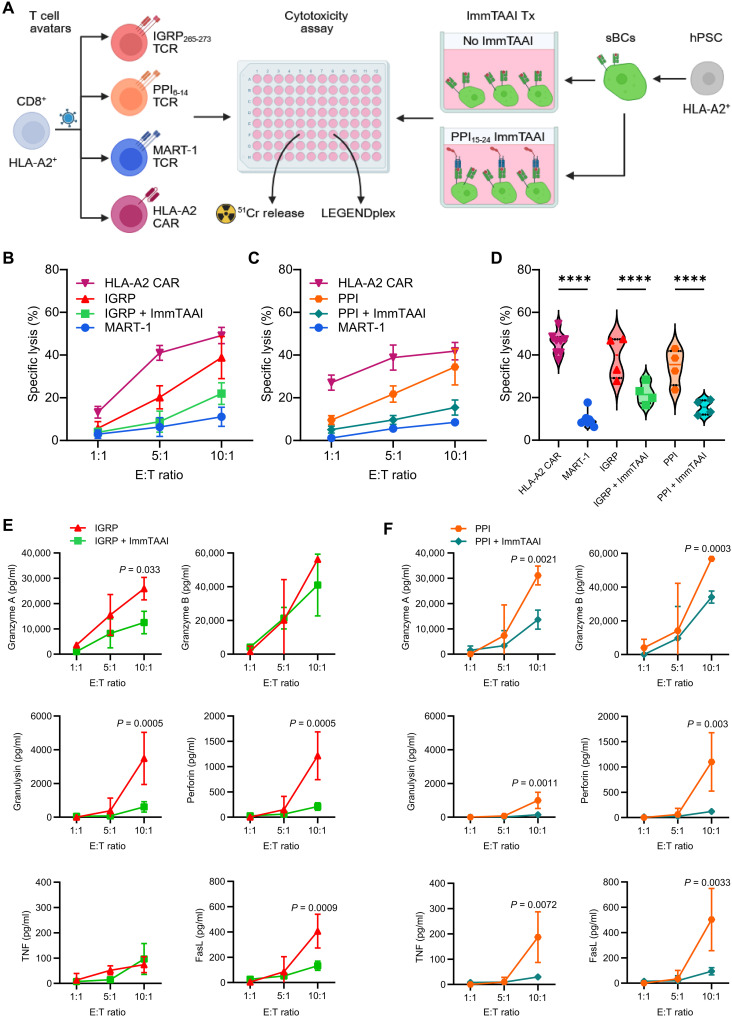
PPI ImmTAAI suppressed killing of sBCs by T cell avatars. (**A**) Experimental scheme depicts the coculture assay system used to evaluate the immunogenicity of sBC treated with ImmTAAI in the presence of HLA-A2 CAR, MelanA_27–35_–reactive (MART-1), IGRP_265–273_–reactive (clone 32), or PPI_6–14_–reactive (clone 15b) CD8^+^ avatars. Created in BioRender. Phelps, E. (2026) https://BioRender.com/n60mjw7. (**B**) Specific lysis of sBCs by IGRP avatars after 24 hours of coculture or (**C**) PPI avatars after 48 hours of coculture with or without ImmTAAI treatment at each effector:target (E:T) ratio. *n* = 4. (**D**) Specific lysis for the 10:1 E:T ratio compared across all groups. ****P < 0.0001 by two-way ANOVA with multiple comparisons. *n* = 4. (**E** and **F**) Line plots show cell culture supernatant concentrations of proinflammatory T cell effector molecules and cytokines (granzyme A, granzyme B, granulysin, perforin, TNF, and FasL) produced by T cell avatars. *P* values are reported for paired samples using two-way ANOVA with Bonferroni’s correction for multiple comparisons.

To understand the ability of ImmTAAI molecules to functionally inhibit effector T cells, we implemented a model of T cell–mediated killing. This system consisted of antigen-specific T cell “avatars” generated via lentiviral TCR gene transfer to primary human CD8^+^ T cells (*49*–*51*) and human stem cell–derived beta cells (sBCs) differentiated from an HLA-A2–positive line to serve as target cells (Fig. 6A) (*52*, *53*). We generated two different T cell avatars specific to beta cell antigens islet-specific glucose-6-phosphatase catalytic subunit related protein (IGRP_265–273_) and PPI_6–14_. These TCRs target different peptides than ImmTAAI molecules (PPI_15–24_), ensuring that downstream functional assays specifically test PD-1 engagement rather than simply masking TCR-pHLA interactions. We also generated a negative control avatar to determine the background activity of the T cell avatars using a TCR specific for Melan-A_27–35_ (also known as MART-1) (*54*), an antigen not expressed in beta cells. Furthermore, we generated a positive control avatar expressing an HLA-A2–specific chimeric antigen receptor (HLA-A2-CAR) that directly recognizes HLA class I on target cells (*55*) to determine the maximal activity of the avatars.

We validated the activation of T cell avatars by kinetic analysis of CD69 and PD-1 up-regulation following stimulation with anti-CD3/anti-CD28 Dynabeads (fig. S4). We next verified that PPI ImmTAAI natively bound to the sBCs without need for exogenous peptide loading (fig. S5). We then cocultured the T cell avatars and sBCs at varying effector to target ratios in the presence or absence of 20 nM PPI ImmTAAI. We performed a chromium release assay to determine the impact of PPI ImmTAAI on the cytotoxicity of the T cell avatars (Fig. 6, B and C). To perform this assay, target beta cells were loaded with radioactive chromium-51 (^51^Cr). When the target cells are lysed by immune effectors, the ^51^Cr is released. The amount of ^51^Cr in the media is compared to a spontaneous release control (target cells alone) to determine the cytotoxic activity of the effector cells (% specific lysis). Specific lysis of sBCs by IGRP- and PPI-specific avatars correlated with the number of T cell avatars and was similar to the HLA-A2-CAR–positive control at the highest effector to target ratio, while T cells specific to the negative control antigen MART-1 demonstrated significantly less sBC killing (Fig. 6, B to D). The small number of sBCs destroyed by MART-1–specific T cells is most likely attributable to allorecognition by the endogenous TCR still present in the T cell avatars. ImmTAAI molecules significantly reduced specific lysis by both IGRP- and PPI-specific T cell avatars (Fig. 6, B to D).

Multiplex cytokine assays were performed on the supernatants from the T cell avatar–sBC coculture experiments (*56*). PPI ImmTAAI treatment significantly down-regulated the expression of an array of effector molecules produced by cytotoxic T cells, including granzyme A, granulysin, perforin, and FasL for IGRP-specific avatars (Fig. 6E) and granzyme A, granzyme B, granulysin, perforin, tumor necrosis factor (TNF), and FasL for PPI-specific avatars (Fig. 6F). Together, these results demonstrate that ImmTAAI effectively protects against beta cell killing by CD8^+^ T cells expressing islet autoreactive TCRs.

### PPI ImmTAAI suppresses killing of primary islets in human pancreas slices by T cell avatars

**Fig. 7.**
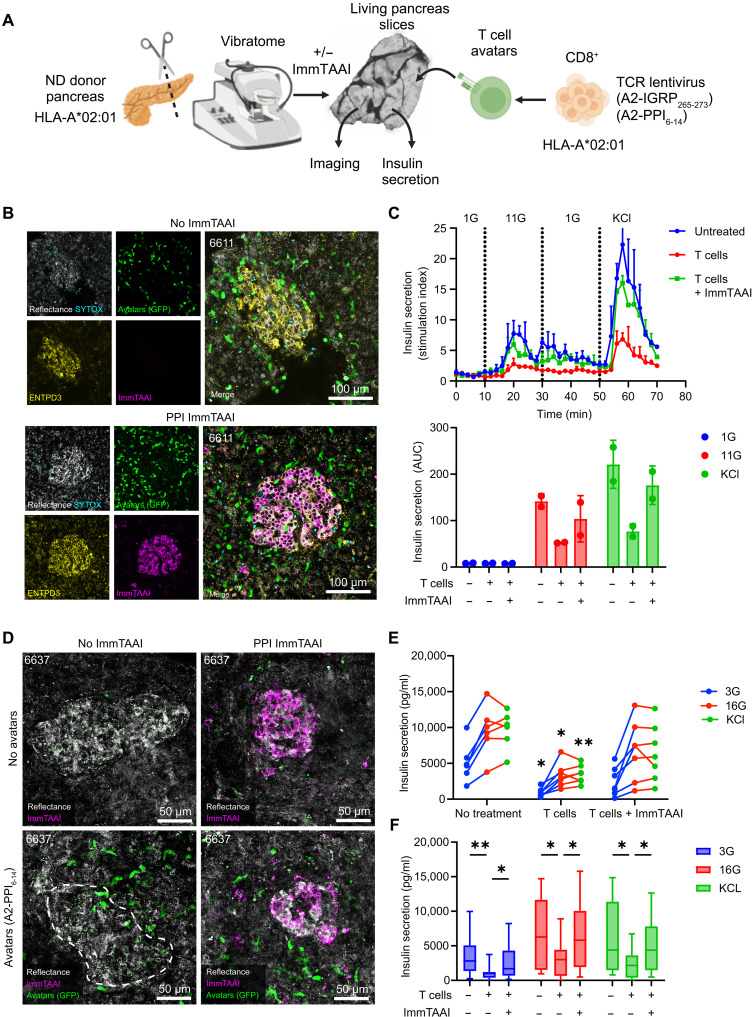
PPI ImmTAAI suppressed killing of beta cells in slices by T cell avatars. (**A**) Experimental scheme depicts the coculture assay system used to evaluate the immunogenicity of pancreas slices treated with PPI ImmTAAI in the presence of IGRP_265–273_–reactive (clone 32), or PP_I6–14_–reactive (clone 15b) CD8^+^ avatars. Created in BioRender. Phelps, E. (2026) https://BioRender.com/rsp2cin. (**B**) Live-cell confocal microscopy images 18 hours after addition of 200,000 IGRP-specific T cell avatars per slice. (**C**) Dynamic perifusion-based glucose and KCl-stimulated insulin secretion and area-under-the-curve (AUC) quantification of slices 48 hours after the addition to IGRP-specific T cell avatars with and without PPI ImmTAAI treatment (20 nM). *n* = 2 perifusion chambers with three slices per chamber; means ± sem. (**D**) Live-cell confocal microscopy images 18 hours after the addition of 200,000 PPI-specific T cell avatars per slice. (**E**) Static glucose and KCl-stimulated insulin secretion from slices 48 hours after the addition to PPI-specific T cell avatars with and without PPI ImmTAAI treatment (20 nM). *n* = 6 to 7 slices per donor. Data are representative of experiments conducted in three donors. (**F**) Combined static glucose and KCl-stimulated insulin secretion from three donors. Means ± range. Statistical differences were determined by two-way ANOVA followed by Tukey’s post hoc analysis. *P < 0.05, **P < 0.01.

Human pancreas slices with T cell infiltration of islets are rare specimens, and we were unable to clearly observe beta cell killing by endogenous immune cells in human pancreas slices from recent-onset T1D donors. We speculate this is because the process of beta cell loss in human T1D occurs over a prolonged period of months to years (*57*), while we are only able to maintain pancreas slices in culture for several days. To further evaluate ImmTAAI molecules’ ability to protect beta cells from T cell killing in the human pancreas tissue, we adapted the in vitro cell–mediated killing assay (Fig. 6A) (*49*, *50*, *58*) into an ex vivo model of T1D by coculturing T cell avatars specific for beta cell antigens (IGRP_265–273_ and PPI_6–14_) with pancreas slices from HLA-A2–positive donors without diabetes (Fig. 7A and table S1). Slices were incubated with 200,000 T cells per slice, with or without 20 nM PPI ImmTAAI, and then evaluated by live imaging for infiltration of islets by T cell avatars and by functional assays for insulin secretion. After 18 hours of coculture, slices showed strong infiltration of ENTPD3^+^ islets by enhanced green fluorescent protein (eGFP) reporter–expressing IGRP_265-273_–specific T cell avatars and strong labeling of ENTPD3^+^ islets with PPI ImmTAAI (Fig. 7B). Perifusion of slices after 48 hours of coculture with IGRP-specific avatars showed reductions in glucose- and KCl-induced insulin secretion with ImmTAAI partially rescuing functional beta cell secretion (Fig. 7C); however, the differences were not tested for statistical significance due to a low sample size because of the need to pool multiple slices per perifusion chamber to reach the detection range of the insulin enzyme-linked immunosorbent assay (ELISA) kit. To improve sample size and throughput, we shifted to testing insulin secretion in single slices using static glucose and KCl incubation. We also shifted to using the PPI_6–14_–specific T cell avatars. As before, we observed eGFP reporter-expressing T cell avatars interacting with islets in slices, with strong labeling of islets with PPI ImmTAAI after the first 18 hours of coculture (Fig. 7D). After 48 hours of coculture, the T cell avatars caused a significant reduction in baseline insulin secretion in 3 mM glucose and significant loss of insulin secretion following 16 mM high glucose and KCl stimulation, while ImmTAAI-treated slices retained their function (Fig. 7, E and F). These observations were consistent across pooled data from repeat experiments in three nondiabetic HLA-A2–positive slice donors (nPOD 6637, 6639, and 6640; table S1) (Fig. 7F).

These results establish coculture of pancreas tissue slices with T cell avatars as an approach to model T1D in tissues from donors without diabetes that creates a condition of autoreactive T cell infiltration of the pancreatic islets and exocrine tissue alongside functional impairment of beta cells. We used this model as an approach to test ImmTAAI as an immunoprotective therapy and showed that it preserves insulin secretory function.

## DISCUSSION

This study investigated the potential for PD-1 agonist ImmTAAI molecules to bind target pHLA in human pancreata and protect against islet autoreactive T cell activity. It was previously shown that cell surface binding of PD-1 agonist ImmTAAI is essential for its activity, as only target cell-bound ImmTAAI molecules can inhibit T cell cytokine secretion and target cell killing (*29*). However, since both the PD-1 agonist nanobody and TCR targeting domain of the ImmTAAI are human specific, the reagents are not compatible with studies in mice. Here, we used pancreas slice technology to characterize ImmTAAI binding in primary human pancreatic tissue.

Live pancreas tissue slices have been used to study islet biology, beta cell dysfunction, and diabetes pathophysiology (*30*, *31*, *33*, *35*, *59*–*61*). Although slices are not a perfect model of the in vivo environment, they offer many advantages over isolated islets (*62*). Our results provide valuable insight into the potential for beta cell–specific ImmTAAI molecules to bind to the intended target cell in heterogeneous, complex tissue environments such as those found in vivo. We showed that PPI ImmTAAI molecules bind specifically to beta cells in a dose-dependent manner. Labeling ImmTAAI with a CF647 fluorophore did not affect its binding kinetics or potency in cell-based assays, providing confidence that the binding we observed in tissue slices accurately reflects what would occur with unlabeled ImmTAAI.

**Fig. 8.**
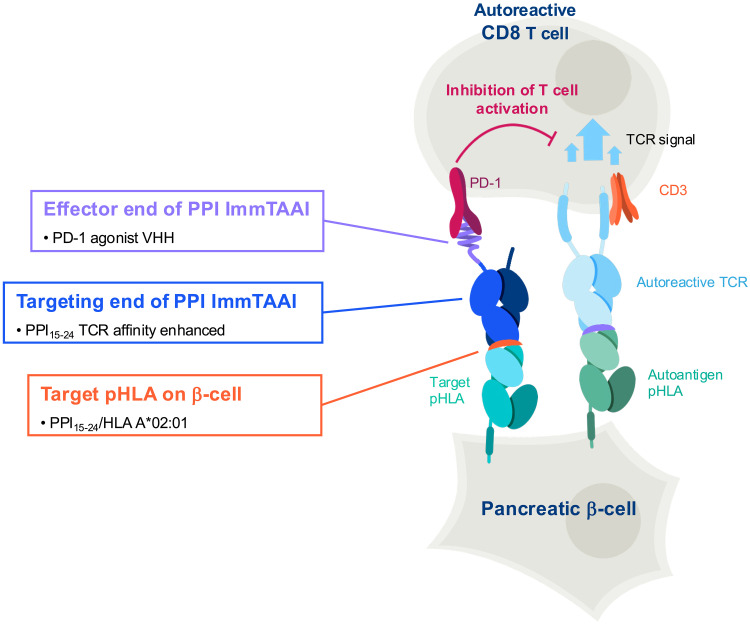
Schematic of ImmTAAI mechanism of action. The TCR targeting domain of ImmTAAI binds to PPI_15–24_ peptide-HLA class I presented by beta cells. The PD-1 agonist effector domain inhibits T cell function and prevents beta cell killing. This mechanism is independent of the TCR specificity of the engaged cytotoxic T cell.

A key piece of data presented here demonstrates ImmTAAI binding in a pancreas actively undergoing autoimmune attack as nPOD donor 6578 had a substantial number of islets with active insulitis defined as at least three islets per pancreas section with ≥15 CD45^+^ cells or ≥6 CD3^+^ T cells immediately adjacent to or within the islet (*63*, *64*), at the time of organ collection, as documented on the nPOD histopathology database (https://npod.org/for-investigators/online-pathology-information/). It is likely that pancreata with active autoimmunity and remaining beta cell mass can bind significant amounts of ImmTAAI since HLA class I hyperexpression on beta cells is a characteristic feature of T1D (*65*–*67*). In addition, inflammation causes increased beta cell PD-L1 expression (*68*, *69*), and we showed previously that PD-1 agonist ImmTAAI molecules are additive with cellular PD-L1 in triggering PD-1 activity on T cells (*29*). This evidence, together with the data shown here, suggests that targeting HLA-A2 presented PPI_15–24_ with the ImmTAAI molecule holds potential for inducing immune tolerance toward beta cells by decreasing CD8^+^ T cell–mediated destruction through activation of the PD-1 pathway in islet-infiltrating T cells (Fig. 8).

Although it is critical to perform studies in T1D and AAb^+^ pancreas donors, these donors are limited and heterogeneous. Thus, we devised an approach to carry out studies in human tissue in which we can control the timing and degree of autoimmunity. To achieve this, we simulated the autoimmune environment of T1D by introducing engineered T cell avatars into living pancreas slices from histocompatible nondiabetic donors. The T cell avatars expressed TCRs specific to beta cell antigens IGRP and PPI. We found that the presence of the islet antigen–reactive T cells was sufficient to cause beta cell dysfunction and/or loss. ImmTAAI treatment preserved beta cell function in this model system, further supporting its future clinical evaluation.

### Limitations of the study

The sparse nature of pancreatic organ donor material limits our capacity to obtain living pancreatic slices, including the need for HLA matching to ImmTAAI molecules and T cell avatars. The functional measurements conducted in slice cocultures with T cell avatars were not directly quantified for the number of surviving beta cells. Although slices contain intact vasculature, this vasculature is not perfused during our experiments. Thus, the cues and mechanisms by which T cells enter slice tissues may differ from that occurring in vivo. Although HLA-A2 is one of the most frequent major histocompatibility complex class I alleles among patients with T1D (*70*), the specific PPI_15–25_ ImmTAAI molecule described in this study would require modification with a different TCR targeting domain to be effective for other HLA alleles.

### Translation to the clinic

Our approach has the advantage of being a soluble protein that is engineered to be highly specific for beta cells through the paired TCR structure and has no activity when unbound (*29*). This class of bispecific, tissue-targeted drugs has recently seen success in cancer therapy, with tebentafusp, a first-in-class TCR-CD3 bispecific therapy, which targets a gp100-derived peptide (expressed in melanocytes and melanoma cells) presented on HLA-A*02:01 molecules. In a phase 3 trial of patients with HLA-A*02:01 and metastatic uveal melanoma (NCT03070392), tebentafusp, which is intravenously administrated on a weekly basis, showed improved clinical benefit with a 1-year survival rate of 73% compared with 59% with investigator’s choice of therapy, thereby receiving US Food and Drug Administration approval in 2022 (*71*). The 3-year follow up analysis of this phase 3 trial continued to show a long-term overall survival benefit of tebentafusp (*72*).

The next step for translation of this class of tissue-targeted bispecific PD-1 agonist is a safety, dosing, and efficacy trial to preserve endogenous beta cell mass in T1D without the need for systemic immune suppression. To maintain ImmTAAI-dependent protection of the pancreatic β-cell microenvironment from immune attack, continuous exposure to the PPI ImmTAAI may be required, unless prolonged immunosuppression of autoreactive T cells may lead to their apoptosis (*73*). In the clinical setting, it will be important to determine whether a shift in autoreactive T cell phenotype toward an unresponsive or exhausted state can be achieved or their numbers are decreased on treatment, and whether this correlates with benefit in patients with T1D.

## MATERIALS AND METHODS

### Research objectives

The objectives of this study were to determine whether ImmTAAI molecules specifically bind to beta cell surfaces in native tissue environments and whether ImmTAAI molecules inhibit cellular immunity in T1D.

### Research subjects or units of investigation

The subjects of this study were organotypic slices from human pancreas and cultured cells.

### Experimental design

Controlled laboratory experiments were performed with PPI ImmTAAI compared to off-target Tel ImmTAAI and untreated samples. Measurements of ImmTAAI molecule localization were by confocal microscopy, and T cell effector function and target cell killing were by in vitro biochemical assays.

### Randomization

Individual pancreas slices were assigned to treatment conditions randomly.

### Blinding

Researchers were blinded to the treatment condition during confocal image acquisition and image quantification. In vitro biochemical assays were performed unblinded but in multiwell plate format where all samples were treated the same.

### Sample size

For human pancreas slices, sample size was determined by the availability of HLA-A2^+^ donor organs, which are intrinsically limited in number, particularly for at-onset T1D donors. Studies in human tissues were validated by parallel in vitro assays with cells/cell lines.

### Rules for stopping data collection

Data were collected for the total available tissue donors that met our selection criteria over the years 2021 to 2025 in accordance with the contract agreement between Immunocore and the University of Florida.

### Data inclusion/exclusion criteria

Currently, nPOD sets organ selection criteria for recovery of pancreata from T1D donors who are younger than 30 years of age and with T1D duration ≤7 years. These selection criteria are based on years of data that residual insulin-positive islets and insulitis are more likely to be found using these acceptance criteria. Control donors are defined as BMI <30, aged 0 to 40 years with no diagnosis of diabetes. Donors were excluded from the study if the slices had low viability (greater than 10% dead cells) or poor islet function (insulin secretion stimulation index less than 2, except for T1D donors, which are expected to have poor islet function). All donors in the study expressed HLA-A2, except for the negative controls for HLA type.

### Outliers

No outliers were excluded from the data.

### Selection of end points

This is not applicable to this study.

### Replicates

In vitro ImmTAAI activity experiments with cell lines (Figs. 1 and 4) were repeated three times. Experiments to optimize the binding conditions (concentration and incubation time) for ImmTAAI molecules in human pancreas slices were conducted using multiple slices from one or two donors per optimization parameter (ImmTAAI TCR specificity, concentration, incubation time, and donor HLA-type) (Figs. 2 and 3 and fig. S1), while ImmTAAI molecule binding at the optimal conditions were repeated in 16 donors (table S1). ImmTAAI activity in T1D pancreas was conducted in a single, rare HLA-A2^+^ organ donor with at-onset T1D and active insulitis (nPOD 6578) (Fig. 5). In vitro ImmTAAI cell killing experiments with T cell avatars (Fig. 6) were repeated twice using two different sBC stem cell lines. ImmTAAI activity to preserve islet function in nondiabetic tissue with the addition of T cell avatars was performed in a single HLA-A2^+^ pancreas for dynamic insulin secretion (6611) and three donors for static insulin secretion (6637, 6639, and 6640) (Fig. 7).

### ImmTAAI production

PPI ImmTAAI, PPI TCR alone and PPI TCR fused to a VHH antibody–specific ACTL-8 were expressed as a soluble molecule in ExpiCHO-S cells (Thermo Fisher Scientific) and purified using three stages of chromatography: anion, cation, and size exclusion (*29*). A control molecule, which binds pHLA from the tumor-associated hTERT (hTERT_540–548_) that is not presented by human cancerous or normal cells (*74*) (Tel ImmTAAI), was expressed as inclusion bodies in *Escherichia coli* followed by refolding and purification using anion and size exclusion chromatography (*75*). CF647 succinimidyl ester labeling was performed according to the manufacturer’s recommendations (Biotium).

### SPR kinetic analysis

SPR binding analysis was performed using a BIAcore 8 K (GE Healthcare) as described previously (*29*). Briefly, biotinylated pHLA-A2 complexes were immobilized on a streptavidin-coated CM5 chip. PPI ImmTAAI, PPI ACTL-8, and PPI TCR were injected over the immobilized pHLA using single-cycle kinetics to assess pHLA affinity. Assessment of PD-1 binding was performed using pHLA-immobilized ImmTAAI followed by injection with PD-1. Data were analyzed using BIAanalysis software (Biacore Insight Evaluation software version 3.0.12.15655, GE Healthcare).

### Cellular TCR signaling assay using the Jurkat NFAT luciferase PD-1 reporter

The ECN90 Jurkat NFL Mel5 PD-1 reporter assay was performed as described previously (*29*). ECN90 cells were plated at 50,000 cells per well in Optiβ3 media into a white 96-well culture plate precoated with β-coat and incubated for 16 to 20 hours at 37°C, 5% CO_2_. Melan-A_26–35_ peptide (ELAGIGILTV heterolytic peptide) was added for 2 hours followed by titrations of ImmTAAI molecules. The assay was initiated by adding 50,000 Jurkat NFL Mel5 PD-1 effector cells and incubated for 16 to 20 hours. For assessment of selectivity, ECN90 cells were replaced with NCI-H1703 cells pulsed with 5 μM Melan-A peptide and treated in an identical fashion. The reporter assays were developed by adding BioGlo reagent (Promega) with NFAT activity determined by measuring luciferase luminescence on a plate reader (Clariostar, BMG Labtech). NFAT activity was normalized against TCR-stimulated controls, with ImmTAAI dose response data analyzed in GraphPad Prism using a four parameter, nonlinear least squares fit to determine IC_50_ values.

### Pancreatic organ donors

Pancreas tissue slices were obtained through the nPOD (RRID: SCR_014641, Gainesville, Florida; https://npod.org/) from donors without diabetes (*n* = 10), donors who were single glutamic acid decarboxylase autoantibody positive (AAb^+^) without diabetes (*n* = 3), and donors with recent-onset T1D (*n* = 2). All procedures using human slices were performed according to the established standard operating procedures (SOP) of the nPOD Organ Processing and Pathology Core (OPPC) as approved by the University of Florida Institutional Review Board (IRB201600029) and the United Network for Organ Sharing (UNOS) according to federal guidelines, with informed consent obtained from each donor’s legal representative. For each donor, a medical chart review was performed and C-peptide measured, with T1D diagnosed according to the guidelines established by the American Diabetes Association (ADA). Demographic data, hospitalization duration, and organ transport time were obtained from hospital records. Donor pancreata were recovered, placed in transport media on ice, and shipped via organ courier to the University of Florida. The tissue was processed by a licensed pathology assistant. nPOD tissues used for this project were approved as nonhuman by the University of Florida IRB (NH00041892 and NH00042022).

### Pancreas slice generation and culture

Pancreas tissue slices were generated by nPOD in accordance with published methods (*33*). Pieces of approximately 1 g were obtained from the pancreas body-tail juncture and placed into extracellular solution (ECS) [125 mM NaCl, 2.5 mM KCl, 26 mM NaHCO_3_, 1.25 mM NaH_2_PO_4_, 1 mM MgCl_2_, 2 mM CaCl_2_, 10 mM Hepes, and 3 mM glucose (pH 7.4)] supplemented with aprotinin (25 kIU/ml, Sigma-Aldrich, #A6106). These were cut into smaller tissue blocks, and connective, adipose, and fibrotic tissues were removed. Small tissue blocks of about 0.5 cm^3^ were embedded in low–melting point agarose (3.8%) and mounted on a specimen holder using superglue (90 to 120 centipoise, World Precision Instruments). The holder was connected to the tray of a semiautomatic vibratome (Leica, VT1200S) and filled with cold ECS supplemented with aprotinin. Tissue slicing was performed at a step size of 120 μm, speed of 0.1 mm/s, amplitude of 1 mm, and an angle of 15°. Slices were collected into a 100-mm petri dish containing 3 mM glucose buffer [137 mM NaCl, 5.36 mM KCl, 0.34 mM Na_2_HPO_4_, 0.81 mM MgSO_4_, 4.17 mM NaHCO_3_, 1.26 mM CaCl_2_, 0.44 mM KH_2_PO_4_, 10 mM Hepes, 0.1% bovine serum albumin (BSA), and 3 mM glucose (pH 7.3)] supplemented with aprotinin. Slices were cultured in low-glucose Dulbecco’s modified Eagle’s medium (DMEM) containing 10% fetal bovine serum (FBS), 1:100 antibiotic antimycotic solution (Corning), and aprotinin in an incubator at 24°C, 5% CO_2_.

### Slice fluorescent staining and imaging

Slices were washed twice with prewarmed (37°C) Kreb’s ringer bicarbonate Hepes buffer (KRBH: 115 mM NaCl, 4.7 mM KCl, 2.5 mM CaCl_2_, 1.2 mM KH_2_PO_4_, 1.2 mM MgSO_4_, 25 mM NaHCO_3_, 25 mM Hepes, 0.2% BSA, and 3 mM glucose) and transferred to a μ-Slide 8 well, chambered coverslip (Ibidi) with one slice per well containing 250 μl of prewarmed KRBH with ImmTAAI (0, 2, or 20 nM) and 0.5 μl of Alexa Fluor 594–labeled anti-ENTPD3 at 37°C, 5% CO_2_ for 1 or 2 hours, as indicated, washed with KRBH buffer three times, and then labeled with 1 μM SYTOX Blue viability stain (Thermo Fisher Scientific) and positioned in the chambered coverslips under a slice anchor (Warner Instruments) for imaging on a Leica SP8 or Leica Stellaris confocal microscope.

### Killing and motility assay

EndoC-βH2 mKate red were generated by transducing EndoC-βH2 cells (Human Cell Design) with HLA-A*02:01/β2-microglobulin and mKate red lentivirus (NucLight red, Sartorius) as described (*29*). EndoC-βH2 cells were plated at 5 × 10^4^ cells per well on TPP 96-well plate pretreated with β-coat solution (Human Cell Design) in Ultiβ1 media and incubated over night at 37°C, 5% CO_2_. The cells were pulsed with PPI_6–14_ peptide at 2 μM for 2 hours. ImmTAAI molecules were added at 10 nM and incubated for 2 hours. PPI_6–14_–HLA-A2–specific autoreactive T cell clone 4b PD-1^+^ was labeled with Cell Trace Violet (eBioscience) and added to the plate. Coculture plates were live imaged by Opera Phenix Plus confocal microscope (PerkinElmer) at 37°C and 5% CO_2_. All conditions were performed in triplicate. For the motility assay, the β cells and T cells were plated at 1:4 effector:target ratio and the images acquired with 40× water objective, eight fields per well with iterations every 2.36 min for 2 hours. For the killing assay, the cells were plated at 1:1 effector:target ratio and images acquired using 10× air objective, four fields per well, with iterations every 2 hours for 72 hours. Images were analyzed using Harmony Software (PerkinElmer).

To perform the killing assay with PPI ImmTAAI and control molecules, the EndoC-βH2 cells pulsed with PPI_6–14_ peptide were incubated for 2 hours with the molecules at different concentrations. To initiate the assay, T cell clone 4b PD-1^+^ was added at 5 × 10^4^ cells per well (1:1 effector:target ratio). Cell killing was determined by quantification of EndoC-βH2 Red cell number over time using the IncuCyte S3 imaging system (Sartorius). The number of red nucleus–labeled cells at each time point was normalized to the initial number of objects to take in account variation in cell density in the area visualized. The number of events were acquired in four images and averaged.

### Generation of T cell avatars

Fresh peripheral blood mononuclear cells (PBMCs) were obtained from human leukapheresis–enriched blood of healthy donors (median age: 31.5 years, range 19 to 38 years, *N* = 4, 75% male) purchased from LifeSouth Community Blood Centers (Gainesville, FL, USA). Lentiviral transduction was used to generate HLA-A*02:01–restricted CD8^+^ T cell avatars that recognize islet-specific glucose-6-phosphatase catalytic subunit (IGRP_265–273_) (*51*), Melan-A (MART-1) (*54*), PPI_6–14_ (clone 15b), or express an HLA-A2-CAR (*55*), as previously described (*50*). Briefly, peripheral blood was overlayed onto Ficoll-Paque Plus medium (Thermo Fisher Scientific) for density gradient centrifugation (1200*g*, 20 min). Recovered PBMCs were treated with ammonium-chloride-potassium lysis buffer (Gibco) for 5 min at 4°C before quenching with 1× phosphate-buffered saline (PBS). Naïve CD8^+^ T cells were isolated from the PBMC fraction using the EasySep Human Naïve CD8^+^ T Cell Isolation Kit (STEMCELL Technologies) according to the manufacturer’s instructions.

Naïve CD8^+^ T cells were plated at 2.5 × 10^5^ cells per well in 1 ml of complete RPMI 1640 media (cRPMI; RPMI 1640 media Phenol Red w/o l-glutamine, 5 mM Hepes, 5 mM MEM nonessential amino acids (NEAA), 2 mM glutamax, penicillin (50 μg/ml), streptomycin (50 μg/ml), 20 mM sodium pyruvate, 50 mM 2-mercaptoethanol, 20 mM sodium hydroxide, and 10% FBS, supplemented with rhIL-2 (100 IU/ml) and rhIL-7 (5 ng/ml)]. The cells were stimulated with Dynabeads Human T-Expander CD3/CD28 (Thermo Fisher Scientific) at a 1:1 bead:cell ratio for 48 hours, after which the cells were treated with protamine sulfate (8 μg/ml) and three transducing units per cell of either the IGRP, MART-1, HLA-A2-CAR, or PPI lentiviral vector before spinnoculation (1000*g* × 30 min at 32°C). Cell culture media were changed every 3 to 4 days, and beads were removed on day nine.

After expansion, the cells were cultured in cRPMI supplemented with rhIL-2 (100 IU/ml) for 3 days before enriching for a pure population of successfully transduced, GFP^+^ T cell avatars, using fluorescence-activated cell sorting (FACS), accomplished with a FACSMelody Cell Sorter (Beckton Dickinson).

### Chromium-release assay

^51^Cr-release assays were performed as described (*56*) to assess the cytotoxic capacity of T cell avatars in the presence of ImmTAAI. Briefly, 30,000 sBC were seeded in Cultrex-coated wells in a 96-well plate and pretreated for 2 days with IFN-γ (100 ng/ml) in CMRL media containing 5% N-21 MAX, 1% NEAA, 1% GlutaMAX, heparin (10 μg/ml), 2 mM cysteine, 10 μM zinc, 100 μM beta-mercaptoethanol, 1 μM T3, 10 μM Alk5i II RepSox, VitC (50 μg/ml), 0.1% trace elements A, 0.1% trace elements B, and 1× penicillin-streptomycin. sBCs were loaded with radiolabeled ^51^CrNa_2_O_4_ (Revvity) at an activity of 1.48 × 10^5^ Bq per well for 4 hours in DMEM, 5 mM Hepes, 5 mM MEM NEAA, penicillin (50 μg/ml), streptomycin (50 μg/ml), 0.02% BSA, and 10% FBS, and washed twice with fresh media. Following radiolabeling, sBCs were cocultured with MART-1, PPI, IGRP, or HLA-A2-CAR T cell avatars at 0:1, 1:1, 5:1, and 10:1 effector:target ratios for 48 hours. For cocultures performed in the presence of ImmTAAI, unlabeled ImmTAAI was added at a concentration of 20 nM at the start of the coculture. Following 24 hours of coculture for IGRP avatars or 48 hours of coculture for PPI avatars, the supernatants were removed and transferred into 6 mm–by–50 mm lime glass tubes. The lysates of adherent cells were collected using a 2% SDS wash and transferred into separate tubes. ^51^Cr activity, measured in counts per minute (CPM), was assessed for both fractions on a Wizard 1470 automatic gamma counter (Revvity). The specific lysis of sBCs was calculated as follows

$$\begin{array}{c} \%\text{Specific lysis}\\ =\text{experimental}\;\left[\frac{\left(\text{CPM}\;\text{of supernatant}\right)}{\left(\text{CPM}\;\text{of supernatant}\right)+\left(\text{CPM}\;\text{of lysate}\right)}\right]\\ -\text{Spontaneous}\;\left[\frac{\left(\text{CPM}\;\text{of supernatant}\right)}{\left(\text{CPM}\;\text{of supernatant}\right)+\left(\text{CPM}\;\text{of lysate}\right)}\right] \end{array}$$

### Cytokine multiplex assay

To quantify the production of cytokines by T cells after coculture with sBCs in the presence and absence of ImmTAAI, cell culture supernatants from the cell-mediated lysis assay were used to evaluate interleukin-2 (IL-2), IL-4, IL-6, IL-10, IL-17A, FasL, IFN-γ, TNF, granzyme A, granzyme B, perforin, and granulysin production. Samples were processed using the LEGENDplex HumanCD8/NK Panel Kit (BioLegend), and data were collected on a 5L Aurora spectral flow cytometer. Data were analyzed using the LEGENDplex Data Analysis Software Suite (version 2024-12-24; BioLegend). Dilution factors and analyte detection ranges are described in table S3.

### Coculture of T cell avatars with pancreas slices

Pancreas tissue slices were maintained in DMEM, 10% FBS, aprotinin (25 kIU/ml) from bovine lung (Sigma-Aldrich, #A6106), and 1% antibiotic-antimycotic solution (Corning, #30-004-CI) at 24°C. Slices were transferred to 12-well plates, one slice per well, and equilibrated at 37°C for 1 hour prior in 2 ml of modified slice culture media without phenol red, without aprotinin, and supplemented with rhIL-7 and rhIL-15 at 10 ng/ml. ImmTAAI molecule 20 nM was preincubated for 4 to 5 hours before the addition of T cell avatars. Subsequently, Corning PYREX cloning cylinders (Thermo Fisher Scientific, #0955221) were positioned on top of slices, and 2 × 10^5^ T cell avatars (IGRP specific or PPI specific) were added as a 50-μl cell suspension (in modified slice media) pipetted slowly into the cloning cylinder. After 2 hours of incubation, cloning cylinders were removed, and the coculturing system was incubated for 48 hours with live imaging every 24 hours.

### GSIS of pancreas slices

Perifusion studies were conducted using a Biorep Technologies Perifusion System (Biorep Technologies) as described (*76*). Three slices each were placed into three closed perifusion chambers and connected to the system. Tissue slices were perifused at a flow rate of 100 μl/min at 37°C with DMEM supplemented with sodium bicarbonate (3.2 g/liter), Hepes (1.11 g/liter), sodium pyruvate (0.11 g/liter), 4 mM l-glutamine (pH 7.4), 0.1% BSA, aprotinin (25 kIU/ml) and 1 mM glucose, 11 mM glucose, or 30 mM KCl. The perifusate was collected in 96-well plates at 2-min intervals. Perifusates were stored at −80°C until commercial insulin ELISAs were run (Alpco, # 80-INSHU-CH01). Static glucose-stimulated insulin secretion (GSIS) was performed immediately after the coculturing experiments. GSIS was performed in 250 μl of KRBH supplemented with glucose at 3 mM glucose, 16.7 mM glucose, or 30 mM KCl. Each stimulation was incubated for 1 hour at 37°C sequentially. Each slice was washed once with 500 μl of KRBH before addition of the next stimulation. The stimulation supernatants were collected, centrifuged at 500*g* for 5 min, and stored at −80°C until insulin measurements by ELISA kit (Alpco, # 80-INSHU-CH01).

### High-resolution HLA typing

HLA typing was carried out according to nPOD protocols (*36*). Genomic DNA was extracted from frozen spleen tissue samples of every donor using DNeasy Blood & Tissue kit (Qiagen). The quality and concentration of purified DNA were assessed by spectrometry using an Epoch microplate absorbance reader (Agilent). High-resolution HLA typing was performed at the Autoantibody/HLA Core Facility, Barbara Davis Center for Childhood Diabetes (Aurora, Colorado). Briefly, the specific HLA region to be typed was amplified, labeling the amplicon with Biotin. Amplicon was applied to sequence-specific oligonucleotide-coupled microspheres (One Lambda). After incubation, streptavidin-phycoerythrin was added to the bead/amplicon mixture. Microspheres were visualized using a LabScan3D instrument (Luminex). The HLA alleles were determined by HLA Fusion software (One Lambda, Los Angeles, California).

### Cell lines

The Jurkat NFAT luciferase T cell line (Promega) was maintained in RPMI 1640, 10% FBS, 2 mM l-glutamine, 0.1 mM MEM NEAA, 1 mM sodium pyruvate, penicillin (50 U/ml), streptomycin (50 μg/ml), and hygromycin B (200 μg/ml). The immortalized human pancreatic beta cell lines, ECN90 and EndoC-βH2 (Human Cell Design) were maintained in Optiβ3 and Ultiβ1 media, respectively, in tissue culture vessels precoated with β-coat (Human Cell Design). The NCI-H1703 non–small lung carcinoma cell line (ATCC) was maintained in RPMI 1640, 10% FBS, 2 mM l-glutamine, penicillin (50 U/ml), and streptomycin (50 μg/ml). Cells were maintained at 37°C, 5% CO_2_.

### Lentivirus production and transduction

Lentiviruses containing PDCD1 (PD-1, obtained from Origene, Rockville, Maryland) or the Mel5 TCR specific for Melan A_26–35_ peptide (produced in-house) presented by HLA-A*02 were packaged by transfection of lentiviral vectors with packaging plasmids (pMD2.g, pMDLg/p RRE and pRSV.REV, in-house) into human embryonic kidney 293T cells with TurboFect Transfection Reagent (Thermo Fisher Scientific, Waltham, Massachusetts). Lentiviral particles were collected 48 hours after transfection, filtered through a 0.45-μm filter, and concentrated by centrifugation at 10,000*g* for 16 hours. Pellets were resuspended in growth media and stored at −80°C before use. Lentivirus was added to 1 × 10^6^ exponentially growing Jurkat NFAT luciferase T cells in wells of a 24-well cell culture plate. After 48 to 72 hours, transduced cells were harvested, assessed for expression of transduced genes by flow cytometry and expanded into cell culture flasks.

### ENTPD3 antibody labeling

Human anti-ENTPD3 antibody (#AF4400, R&D Systems, Minneapolis, Minnesota) was reconstituted to 2 mg/ml in sterile 0.1 M NaHCO_3_ buffer and labeled with Alexa Fluor 594 *N*-hydroxysuccinimide ester (Invitrogen, Waltham, Massachusetts) according to the manufacturer’s recommendations. Unbound dye was removed by a Zeba 0.5-ml desalting column with 7-kDa molecular weight cutoff (Thermo Fisher Scientific, Waltham, Massachusetts). Purified, labeled antibody was aliquoted and stored at −20°C until use.

### Image analysis

MFI and colocalization were quantified using Fiji software (ImageJ). MFI of each islet was calculated by subtracting the mean intensity of a background region of interest (ROI). ImmTAAI colocalization was measured using the JaCOP plugin for ImageJ (*77*). Pearson’s coefficient was calculated between ImmTAAI and anti-ENTPD3 within a rectangular ROI comprising approximately 25% of the islet area, drawn to include labeled and unlabeled portions, while avoiding bright autofluorescent artifacts. T cell tracking was performed using the manual tracking mode for the TrackMate plugin for ImageJ (*78*).

### Generation of T cell clones

T cell clones 4b and 15b specific for the pancreatic β-cell antigen PPI_6–14_ (HLA-A*02:01 RLLPLLALL; 4b clone TCR has a dissociation constant (*K*_d_) ~800 μM, 15b clone TCR has a *K*_d_ ~48 μM) were generated as described (*29*). T cell clone specificity was validated by dextramer staining (made in-house from biotinylated PPI_6–14_ pHLA-A2 and fluorescently labeled Streptavidin-Dextramer, Immundex ApS) and specific killing of EndoC-βH2 HLA-A*02 cells pulsed with PPI_6–14_ peptide. The T cell clone 4b was transduced with lentivirus containing PDCD1 (PD-1) (OriGene, #RC210364L1) as described (*29*). The clone was expanded with allogeneic irradiated PBMCs from three donors in the presence of PHA-L (1 μg/ml) and IL-2 (100 U/ml) in T cell cloning media (RPMI 1640, 5% human AB serum, 1% penicillin-streptomycin, 1% l-glutamine, 1% MEM NEAA, and 1% sodium pyruvate).

### Flow cytometric assessment of avatar activation capacity

The baseline activation status and capacity for T cell activation of the IGRP-reactive CD8^+^ T cell avatars were established following 12 days of culture. Using approximately 1 × 10^6^ cells per condition, the cells were stimulated with Dynabeads Human T-Expander CD3/CD28 at a 1:1 bead:cell ratio for either 0, 24, 48, or 72 hours. Following culture, avatars underwent viability staining with Live/Dead Near-IR viability dye (Thermo Fisher Scientific) for 10 min at 4°C before washing with stain buffer (PBS + 2% FBS + 0.05% NaN_3_ w/v). Before staining, the cells were treated with TruStain FcX (BioLegend) for 5 min at 23°C then stained with an extracellular antibody cocktail consisting of CD8-Alexa Fluor 700, CD69-BV421, and CD279-BV650 (clone and manufacturer information provided in table S2) with Brilliant Stain Buffer Plus (Beckton Dickinson Biosciences) for 30 min at 4°C. Data were collected on an Aurora 5L (16UV-16 V-14B-10YG-8R) spectral flow cytometer (Cytek, Fremont, CA, USA), and analysis was conducted using FlowJo version 10.8.1 Software (BD Life Sciences).

### Human stem cell culture and sBC differentiation

Undifferentiated human pluripotent stem (hPSC) Mel1^INS-GFP^ reporter cells (*79*) were maintained on hESC qualified Cultrex (Biotechne, #3434-005-002) in mTeSR^+^ media (STEMCELL Technologies, #05826). Differentiation to sBCs was carried out in suspension-based, magnetic stirring platforms (Reprocell, #ABBWVS03A-6, #ABBWVDW-1013, #ABBWBP03N0S-6) as described (*80*, *81*). Briefly, 90% confluent hPSC cultures were dissociated into single-cell suspension by incubation with TrypLE (Gibco, #12-604-021). Dissociation was halted with mTeSR^+^ media, and cells were counted using a Countess 3 cell counter (Thermo Fisher Scientific), followed by seeding 0.5 × 10^6^ cells/ml in mTeSR^+^ media supplemented with 10 μM ROCK inhibitor in spinner flasks. Three-dimensional sphere formation was performed for 48 to 72 hours. Differentiation media were changed daily by letting spheres settle by gravity for 3 to 5 min. Most supernatant was removed by aspiration; fresh media were added, and spinner flasks were placed back on stirrer system. sBC differentiation and cryopreservation were based on our published protocol (*52*, *82*) with modifications as outlined below. Differentiation media are as follows: induction of definitive endoderm differentiation using day 1 (d1) media [RPMI containing 0.2% FBS, 1:5000 insulin-transferrin-selenium (Gibco, #41400-045), activin A (200 ng/ml; R&D Systems, #338-AC-01 M), and 3 μM CHIR99021 (STEMCELL Technologies, #72054)]; d2 and d3: RPMI containing 0.2% FBS, 1:2,000 ITS, and activin A (100 ng/ml); d4 and d5: RPMI containing 2% FBS, 1:1000 ITS, and Human FGF-7 (KGF) (50 ng/ml; Prepotech, #100-19-1MG); d6 and d7: DMEM with d-glucose (4.5 g/liter; Gibco, #11960-044) containing 1:50 N-21 MAX (Biotechne, #AR008), 1:100 NEAA (Gibco, #11140-050), 1 mM sodium pyruvate (Gibco, #11360-070), 1:100 GlutaMAX (Gibco, #35050-061), 3 nM 4-[(E)-2-(5,6,7,8-tetrahydro-5,5,8,8-tetramethyl-2-naphthalenyl)-1-propenyl]benzoic acid (TTNPB, Tocris Bioscience, #0761), 250 nM Sant-1 (R&D Systems, #1974), 250 nM LDN (STEMCELL Technologies, #72149), 30 nM phorbol 12-myristate 13-acetate (Sigma-Aldrich, #P1585-1MG), and 2-phospho-l-ascorbic acid trisodium salt (VitC) (50 μg/ml; Sigma-Aldrich, #49752-10G); d8 and d9: DMEM containing 1:50 N-21 MAX, 1:100 NEAA, 1 mM sodium pyruvate, 1:100 GlutaMAX, EGF (100 ng/ml; R&D Systems #236-EG-01M), KGF (50 ng/ml), and VitC (50 μg/ml); d10 to d15: DMEM containing 1:50 N-21 MAX, 1:100 NEAA, 1 mM sodium pyruvate, 1:100 GlutaMAX, heparin (10 μg/ml; Sigma-Aldrich, #H3149-250KU), 2 mM *N*-acetyl-l-cysteine (Cysteine) (Sigma-Aldrich, #A9165-25G), 10 μM zinc sulfate heptahydrate (zinc) (Sigma-Aldrich, #Z0251-100g), 1× BME, 10 μM Alk5i II RepSox (R&D Systems, #3742/50), 1 μM 3,3′,5-triiodo-l-thyronine sodium salt (T3) (Sigma-Aldrich, #T6397), 0.5 μM LDN, 1 μM gamma secretase inhibitor XX (XXi) (AsisChem, #ASIS-0149), and 1:250 1 M NaOH to adjust pH to ~7.4; d16 to d30: CMRL (Gibco, #11530-037) containing 1:50 N-21 MAX, 1:100 NEAA, 1:100 GlutaMAX, heparin (10 μg/ml), 2 mM cysteine, 10 μM zinc, 1× BME, 1 μM T3, VitC (50 μg/ml), 1:1000 trace elements A (Corning # 25-021-CI), 1:1000 trace elements B (Corning, # 25-022-CI), 10 μM Alk5i II RepSox, and 1:250 NaOH to adjust pH to ~7.4. All media, except for mTeSR^+^, also contained 1× penicillin-streptomycin.

### Cryopreservation and thawing of sBC

Day 23 sBCs were dissociated into single cells and cryopreserved as described (*53*, *80*). Briefly, cells were quenched with 2% FBS in PBS and filtered using a cell strainer into FACS 5-ml tubes. Cells were counted using a Countess 3 cell counter (Thermo Fisher Scientific) and resuspended at 3 × 10^6^ cells/100 μl of CryoStor CS10 (STEMCELL Technologies). Cells were cryopreserved overnight before transferring to liquid nitrogen for long-term storage. For thawing, 1 ml of warm sBC media (d16-30 media described above) was added to the thawed cryovial dropwise before the entire volume was transferred into 5 ml of sBC media, counted, and seeded in Aggrewell 800 plates to generate clusters with 3000 cells per cluster. After 24 hours, a partial media change was done to remove any debris. Fully formed clusters were generated after 48 to 72 hours.

### ImmTAAI sBC binding assay

Day 23 sBC cryovials were thawed and plated into Aggrewell plates as described above with or without the presence of 20 nM ImmTAAI for 24 hours. Upon the partial media change at 24 hours, fresh ImmTAAI was added at 2 nM to the ImmTAAI-treated cells or nothing as a control. After a total of 48 hours of reaggregation, half of the clusters were incubated for another 24 hours (total of 72 hours) with a redosing of 2 nM ImmTAAI, while the other half were collected for imaging of ImmTAAI binding via confocal microscopy or dissociated into single cells and run live on the flow cytometer for quantification of ImmTAAI binding at the population level as detailed below. At 72 hours, these analyses were repeated with the additional clusters.

### sBC flow cytometry

For sBC, single-cell suspensions were made by washing clusters with PBS and incubating with 0.05% Trypsin with EDTA at 37°C for 12 to 15 min to create a single-cell suspension. Cells were quenched with 2% FBS in PBS and filtered using a cell strainer into FACS 5-ml tubes. Live cells were resuspended in FACS buffer for analyses on 5-laser Cytek Aurora for the pINS.GFP reporter marking beta cells and the ImmTAAI AF647. Analysis was performed using FloJo software v10.9 (BD Life Sciences).

### Statistical analysis

Sample size (*n*) was defined as the number of separate islets measured per condition. Means among three or more groups were compared by one-way or two-way analysis of variance (ANOVA) followed by Tukey’s post hoc pairwise comparisons using GraphPad Prism 9 software. A confidence level of 95% was considered significant. Statistical test used, exact *P* values, error bars, and definition of *n* are all indicated in the individual figure legends.
