# Supplementary material for: Beta cell–targeted PD-1 agonist inhibits cell-mediated autoimmunity in pancreas tissue slices
Source: Sci Adv. 2026 Apr 1;12(14):eaec9029. doi: 10.1126/sciadv.aec9029 (PMC13041756; doi:10.1126/sciadv.aec9029)
Supplement: Supplementary file 1 — Figs. S1 to S5 Tables S1 to S3 Legends for movies S1 and S2 [file sciadv.aec9029_sm.pdf]

Science Advances
AAAS

Supplementary Materials for

Beta cell–targeted PD-1 agonist inhibits cell-mediated autoimmunity in pancreas tissue slices

Matthew W. Becker *et al.*

Corresponding author: Edward A. Phelps, ephelps@bme.ufl.edu

Sci. Adv. **12**, eaec9029 (2026)
DOI: 10.1126/sciadv.aec9029

The PDF file includes:

Figs. S1 to S5
Tables S1 to S3
Legends for movies S1 and S2

Other Supplementary Material for this manuscript includes the following:

Movies S1 and S2

SUPPLEMENTARY FIGURES

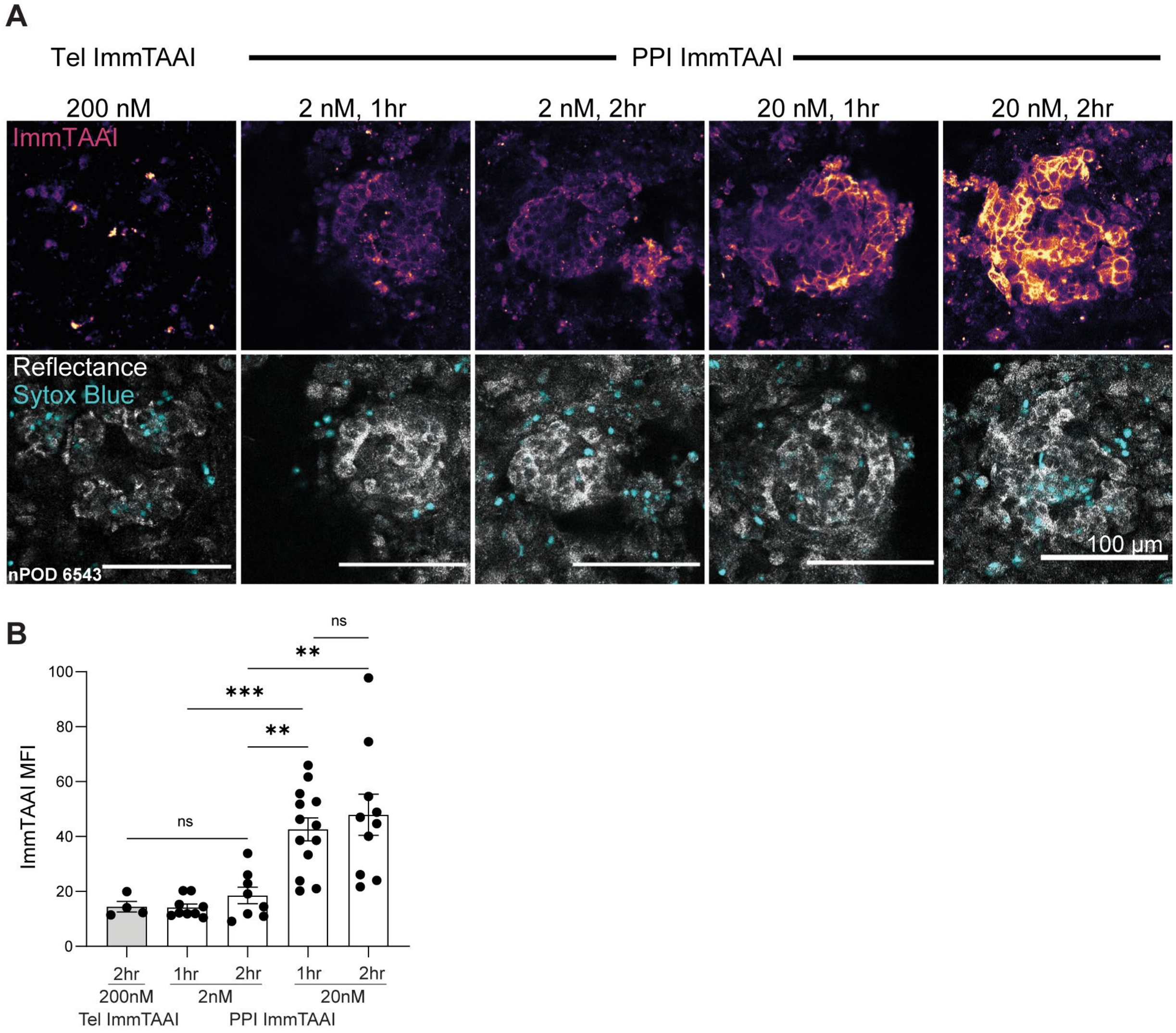

Fig. S1. Optimizing ImmTAAI binding conditions to live pancreatic tissue slices. (**A**) Confocal microscopy images of pancreas slices incubated with CF647-labeled PPI ImmTAAI at 2 or 20 nM for 1 or 2 hours. Slices were also stained with Sytox Blue for viability. (**B**) MFI quantification of microscopy images showing increased PPI ImmTAAI binding at 20 nM, with the highest average MFI at 2 hours. Each dot represents a separate islet from 1 separate slice per condition and is the mean of 2-4 z-planes taken per islet. Statistical differences were determined by one-way ANOVA followed by Tukey's post-hoc analysis. ns = non-significant, ** $p < 0.01$, *** $p < 0.001$.

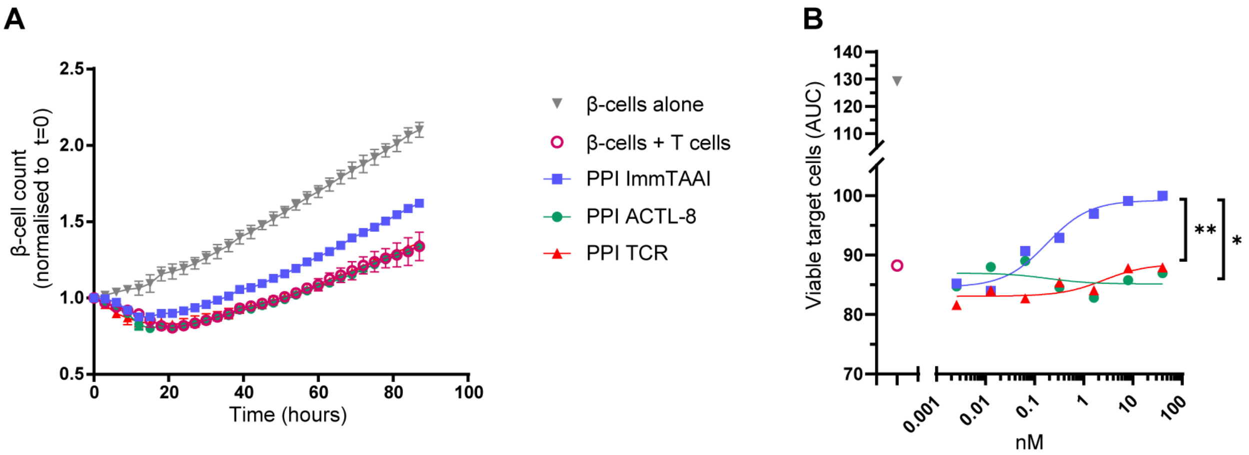

Fig. S2. PPI TCR alone or fused to a VHH antibody anti-ACTL-8 molecule do not suppress T cell-mediated killing of beta cells. (**A**) PPI_{6-14}-HLA-A2-specific autoreactive T cell clone 4b was added to EndoC-β H2 red cells at 1:1 E to T ratio in the presence of PPI ImmTAAI, PPI ACTL-8 or PPI TCR titrations. EndoC-β red target cell number, relative to cells at time 0 (t = 0) at each molecule concentration was measured over time by imaging and growth curves generated. The growth curves measured with 40nM concentration of each molecule are represented. (**B**) Dose-response curves were determined by calculating the AUC of growth curves at different molecule concentrations. Statistical differences were determined by two-way ANOVA followed by Tukey's test. *$p<0.05$, ** $p < 0.01$.

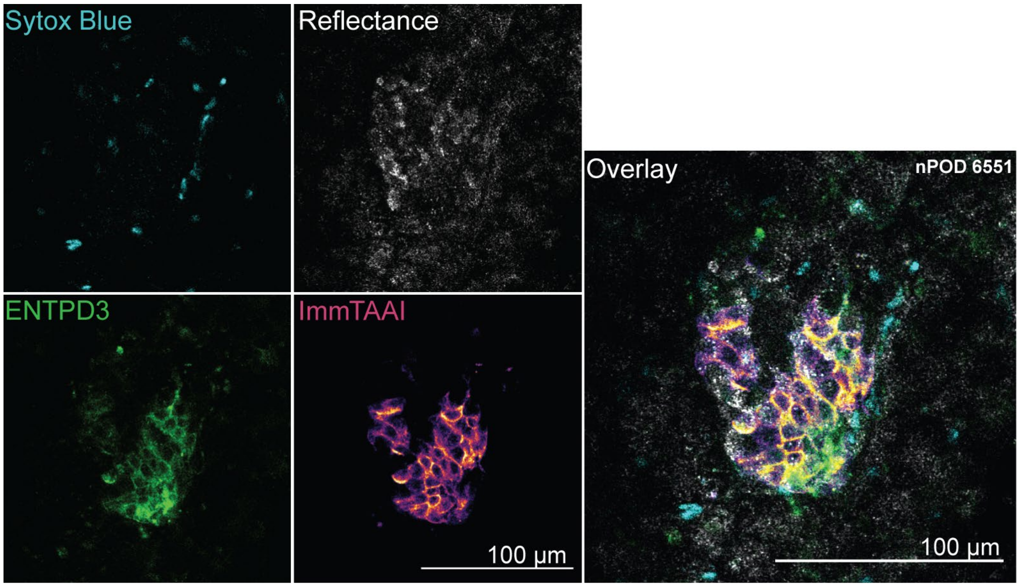

Fig. S3. PPI ImmTAAI binding in a donor with recent diagnosis of T1D and residual beta cells. Live cell confocal microscopy of ImmTAAI binding to ENTPD3^{+} beta cell surfaces in remaining islets in a donor with T1D.

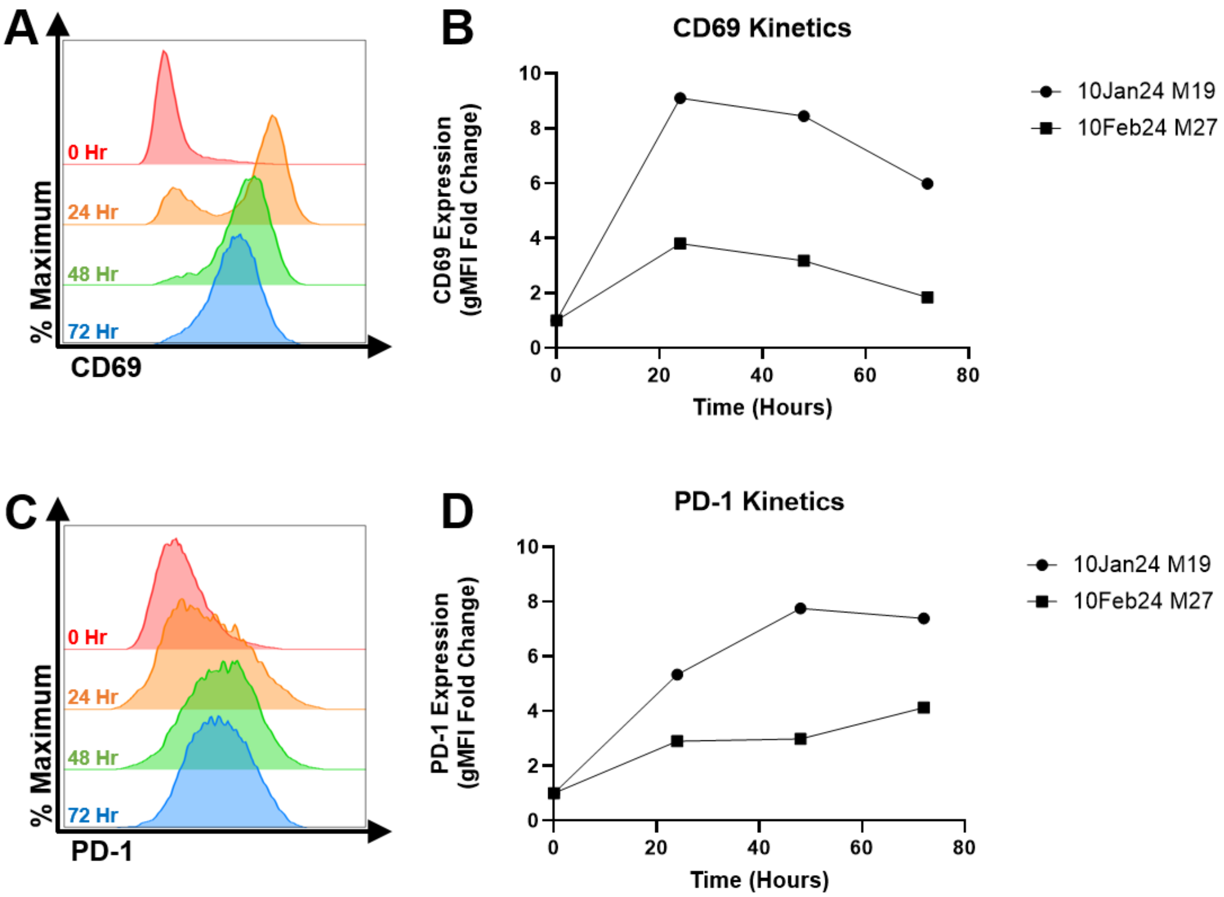

Fig. S4. Assessment of avatar activation capacity. Flow cytometric assessment of (**A-B**) CD69 and (**C-D**) PD-1 expression on expanded IGRP-reactive CD8+ T cell avatars after anti-CD3/CD28 stimulation was used to evaluate functionality. (A, C) Representative histograms show expression of activation markers following 0 (red), 24 (orange), 48 (green), or 72 (blue) hours of stimulation with (B, D) paired dot plots showing the activation marker expression kinetics, relative to baseline. N = 2 biological.

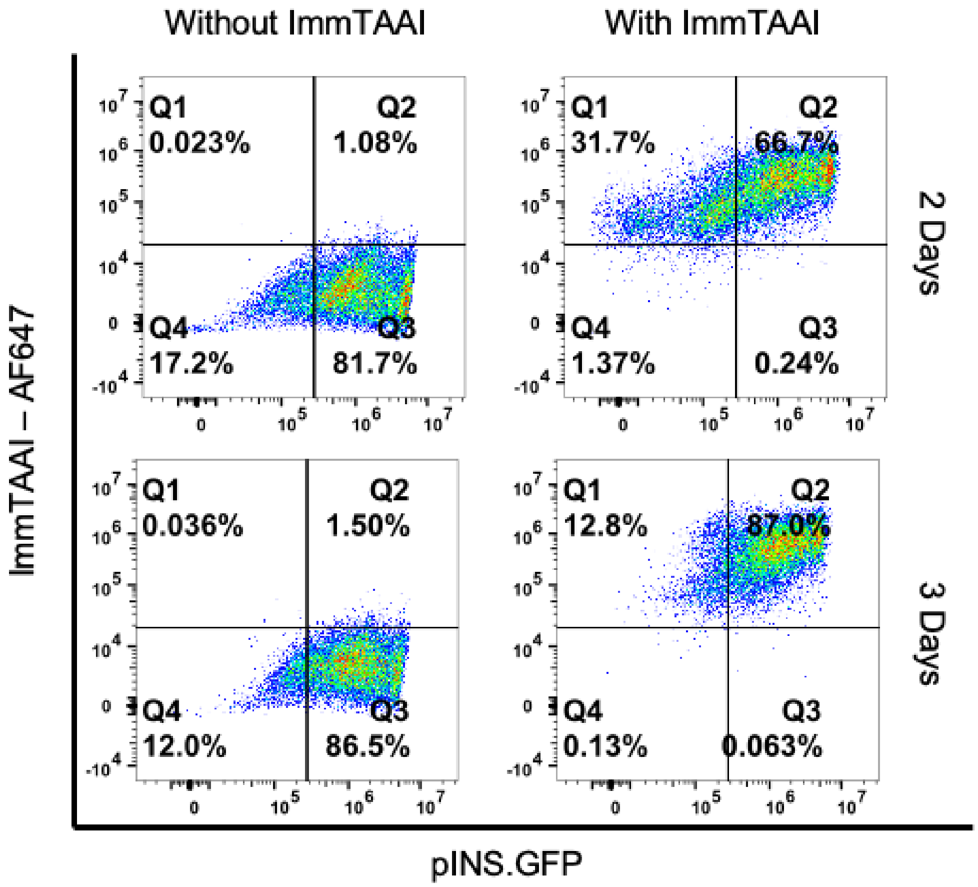

Fig, S5. PPI ImmTAAI binding to stem cell-derived beta cells. Flow cytometric assessment of PPI ImmTAAI binding to stem cell-derived beta cells during reaggregation into clusters on day 2 and day 3 of aggregation. Beta cells express eGFP under the insulin promoter. PPI ImmTAAI added at 20 nM in reaggregation culture media.

SUPPLEMENTARY TABLES

Table S1: Donor information.

nPOD ID	RRID #	Donor Type	AutoAbs	Age (yrs)	Sex	C-pep (ng/mL)	HLA-A	Experiments used
6538	SAMN25652249	Aab+	GADA+	19	Male	11.33	01:01/02:01	Fig 2A
6539	SAMN25652250	No diabetes	Negative	24	Male	39.23	02:01/11:01	Fig 2C-E
6540	SAMN25652251	No diabetes	Negative	6	Female	2.03	29:01/31:01	Fig 3A-C
6543	SAMN25652254	No diabetes	Negative	3	Male	1.45	02:01/31:01	Fig S1
6548	SAMN25652259	No diabetes	Negative	20	Male	4.04	02:01/30:02	Fig 2C
6551	SAMN25652262	T1D (0.5 yrs duration)	GADA+ IA2A+ mIAA+ ZnT8A+	20	Male	0.11	02:01/29:02	Fig S2
6552	SAMN30386842	No diabetes	Negative	33	Female	1.8	31:01/32:01	Fig 3A-C
6553	SAMN30386843	Aab+	mIAA+	12	Female	4.62	02:01/03:01	Fig 3A-C
6575	SAMN33284293	Aab+	GADA+	23	Male	5.21	01:01/02:01	Fig 3F-G
6578	SAMN33284295	T1D (0.0 yrs duration)	IA2A+ ZnT8A+	11	Female	0.35	02:01/03:01	Fig 4A-D
6611	SAMN44486497	No diabetes	Negative	14	Male	10.98	02:01/02:01	Fig 2B, Fig 6B
6615	SAMN44486501	No diabetes	Negative	14	Male	2.9	02:01/68:01	Fig 3D
6637	SAMN49972145	Aab+	GADA+	36	Male	14.36	02:01/29:02	Fig 6D,E
6639	SAMN51857758	No diabetes	Negative	12	Female	9.13	02:01/02:01	Fig 6E
6640	SAMN51857759	No diabetes	Unavailable (no serum)	4	Female	Unavailable	02:01/24:02	Fig 6E

Table S2: Antibodies Used for Flow Cytometry

Target	Clone	Fluorophore	Vendor	Host Species	Concentration	RRID
CD8	SK1	Alexa Fluor 700	BioLegend	Mouse	1.00 µg/mL	AB_2562790
CD69	FN50	BV421	BioLegend	Mouse	1.00 µg/mL	AB_2561909
PD-1	EH12.2H7	BV650	BioLegend	Mouse	1.00 µg/mL	AB_2566362

Table S3: LEGENDplex Detection Ranges

LEGENDplex Assay Supernatant Dilutions		
**Analyte**	**Dilution Factor**	**Adjusted Detection Range (pg/mL)**
IL-17A	1; ND	2.9 to 12,000
IL-2	1; ND	16.6 to 68,000
IL-4	1; ND	3.9 to 16,000
IL-10	1; ND	3.7 to 15,000
IL-6	1; ND	4.6 to 19,000
TNF	1	3.9 to 16,000
Fas	1; ND	14.9 to 61,000
FasL	1	2.4 to 10,000
IFN-γ	100	39 to 160,0,00
Granzyme A	2	12.6 to 52,000
Granzyme B	2	28.2 to 116,000
Perforin	1	2.9 to 12,000
Granulysin	1	11.0 to 45,000
ND = Not Detectable; Lot: B435969		

SUPPLEMENTARY VIDEOS

Video S1. Timelapse confocal recording of an islet in a pancreas slice from T1D donor 6578 with CD3+ T cell infiltrate not treated with PPI ImmTAAI. Recording duration is 30 minutes. SYTOX Blue (cyan), ENTPD3 (yellow), CD3 (green), untreated PPI ImmTAAI channel (magenta). Related to Fig. 5B.

Video S2. Timelapse confocal recording of an islet in a pancreas slice from T1D donor 6578 with CD3+ T cell infiltrate treated with PPI ImmTAAI. Recording duration is 30 minutes. SYTOX Blue (cyan), ENTPD3 (yellow), CD3 (green), PPI ImmTAAI (magenta). Related to Fig. 5B.
